# Enteric infection induces Lark-mediated intron retention at the 5′ end of *Drosophila* genes

**DOI:** 10.1186/s13059-019-1918-6

**Published:** 2020-01-17

**Authors:** Maroun Bou Sleiman, Michael Vincent Frochaux, Tommaso Andreani, Dani Osman, Roderic Guigo, Bart Deplancke

**Affiliations:** 1 Laboratory of Integrative Systems Physiology, Institue of Bioengineering, Ecole Polytechnique Fédérale de Lausanne (EPFL), Lausanne, Switzerland; 2 Laboratory of System Biology and Genetics and Swiss Institute of Bioinformatics, Institute of Bioengineering, Ecole Polytechnique Fédérale de Lausanne (EPFL), Lausanne, Switzerland; 3 Computational Biology and Data Mining Group, Institute of Molecular Biology, Ackermannweg 4, 55128 Mainz, Germany; 4 Faculty of Sciences III and Azm Center for Research in Biotechnology and its Applications, LBA3B, EDST, Lebanese University, Tripoli, 1300 Lebanon; 5 Centre for Genomic Regulation (CRG), The Barcelona Institute of Science and Technology, 08003 Barcelona, Catalonia Spain

**Keywords:** Infection, Gut, Splicing, Intron retention, *Drosophila melanogaster*, DGRP, RBM4, LARK, Systems genetics, sQTL, Small exon

## Abstract

**Background:**

RNA splicing is a key post-transcriptional mechanism that generates protein diversity and contributes to the fine-tuning of gene expression, which may facilitate adaptation to environmental challenges. Here, we employ a systems approach to study alternative splicing changes upon enteric infection in females from classical *Drosophila melanogaster* strains as well as 38 inbred lines.

**Results:**

We find that infection leads to extensive differences in isoform ratios, which results in a more diverse transcriptome with longer 5′ untranslated regions (5′UTRs). We establish a role for genetic variation in mediating inter-individual splicing differences, with local splicing quantitative trait loci (*local*-sQTLs) being preferentially located at the 5′ end of transcripts and directly upstream of splice donor sites. Moreover, *local*-sQTLs are more numerous in the infected state, indicating that acute stress unmasks a substantial number of silent genetic variants. We observe a general increase in intron retention concentrated at the 5′ end of transcripts across multiple strains, whose prevalence scales with the degree of pathogen virulence. The length, GC content, and RNA polymerase II occupancy of these introns with increased retention suggest that they have exon-like characteristics. We further uncover that retained intron sequences are enriched for the Lark/RBM4 RNA binding motif. Interestingly, we find that *lark* is induced by infection in wild-type flies, its overexpression and knockdown alter survival, and tissue-specific overexpression mimics infection-induced intron retention.

**Conclusion:**

Our collective findings point to pervasive and consistent RNA splicing changes, partly mediated by Lark/RBM4, as being an important aspect of the gut response to infection.

## Background

The eukaryotic transcriptome is regulated by diverse mechanisms that ensure robustness and flexibility to adapt to different conditions. Alternative mRNA splicing is one mechanism that contributes to achieving this complex task. Among its postulated functions is an increase in the repertoire of protein-coding genes through the production of multiple isoforms [1]. In addition, mRNA splicing could contribute to (post) transcriptional regulation in that transcript isoforms with the same coding potential can still feature diverse untranslated regions or alternative transcription start sites, which may affect RNA stability and/or translation efficiency [2]. This form of transcriptional regulation can also be affected by external stressors, notably heat shock [3–6], as first shown in *Drosophila* through the accumulation of *Hsp83* and *Adh* pre-mRNAs at severe temperatures [7]. While there are several examples of interactions between splicing and cell stress [3–6, 8], there have been very few genome-wide studies exploring this phenomenon [6].

The *Drosophila* gut has lately attracted a lot of attention in the scientific community as a convenient system to study intestinal homeostasis in normal and diseased conditions [9]. Far from being a simple digestive tube, we now know that it is a highly compartmentalized, dynamic, and immunocompetent organ [10]. Contact with pathogenic bacteria leads to the mobilization of potent immune and stress responses, followed by homeostatic processes, all of which need to be tightly regulated. Several studies have already dissected the transcriptional programs of the innate immune system, demonstrating that a considerable level of gene regulation is achieved through the action of several transcription factors [11]. However, the importance of post-transcriptional regulation in the innate immune response is only beginning to be appreciated [12]. Here, we performed a systematic analysis of alternative splicing in the context of enteric infection in *Drosophila melanogaster*. In addition to classical laboratory strains, we made use of a large RNA sequencing study of 38 inbred lines from the Drosophila Genetic Reference Panel (DGRP) to study this phenomenon in different environmental conditions and genetic backgrounds [13]. Next to characterizing the effect of genetic polymorphisms, we found considerable and reproducible (i.e., genotype-independent) changes in transcript splicing following infection, with a tendency to have more intron retention and thus longer transcripts. Introns with increased retention were overrepresented at the 5′ end of transcripts and were enriched for the RNA binding motif (RBM) of Lark/RBM4. Through knockdown and overexpression of *lark* in adult female enterocytes, we found that *lark* levels can affect intron retention and modulate fly survival after enteric infection. Our work thus provides new insights into the dynamics and importance of the alternative splicing landscape during an innate immune response.

## Results

### Enteric infection leads to extensive changes in transcript isoform ratios

**Fig. 1:**
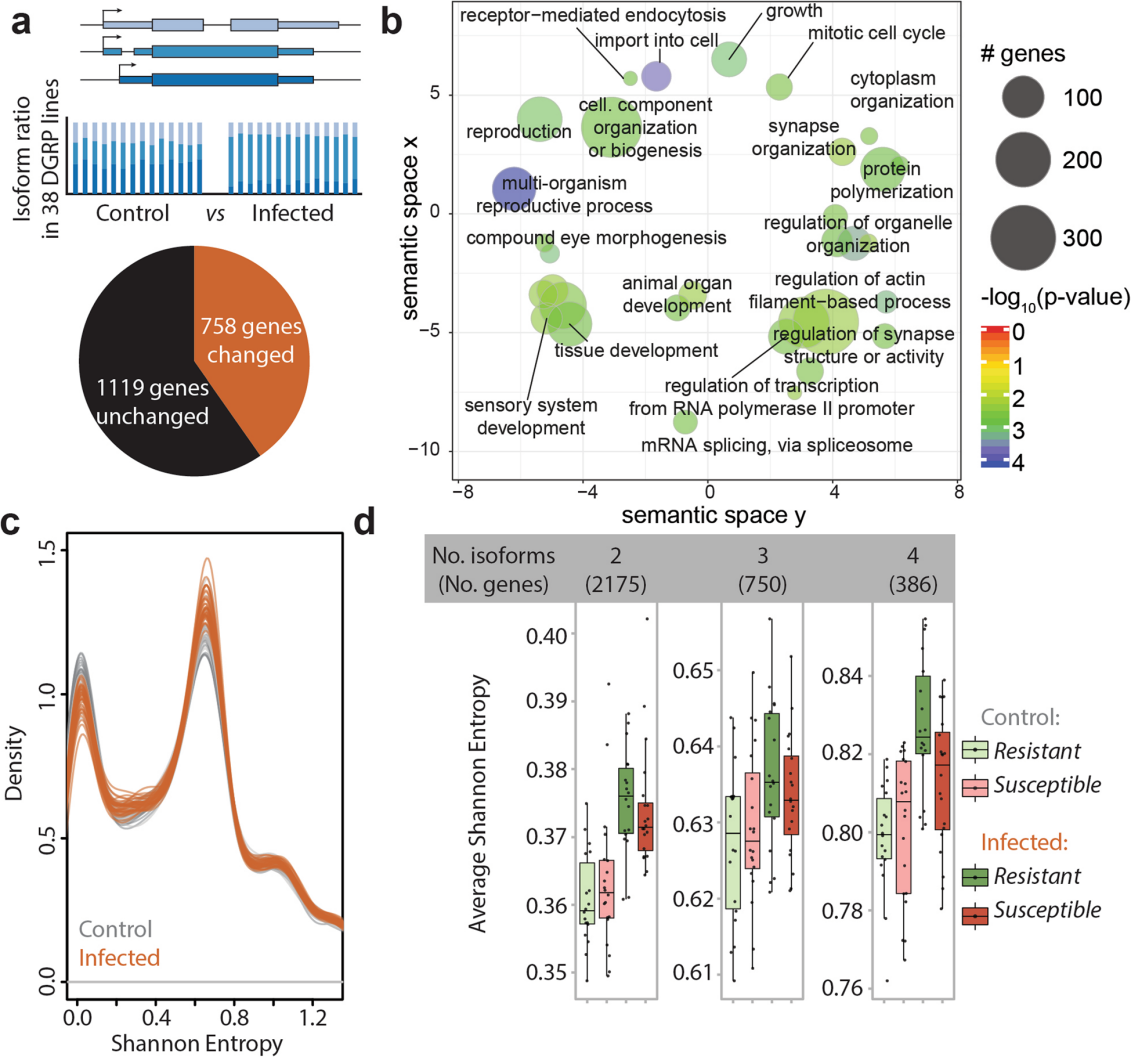
Enteric infection leads to extensive changes in transcript isoform ratios and to greater isoform diversity. **a** Top: schematic illustrating how genes with different isoform ratios are compared between two conditions. MISO [16] was used to calculate the ratios of different annotated isoforms, and thereafter, the rasp package [15] was used to determine significance (p-homogeneity > 0.05, BH adjusted *p* value < 0.05, effect size > 0.1). Bottom: Venn diagram of the number of expressed genes whose isoform ratios were significantly altered after infection. **b** Graphical representation of enriched biological process gene ontology terms based on the list of genes whose isoform ratios were altered after infection. The GO analysis was performed using the GOstats R package [18] (Hypergeometric test *p* value < 0.005), and REVIGO [19] was used to reduce redundancy in the ontology groups and plot them by semantic similarity (allowed similarity = 0.7). The size of each circle indicates the number of genes belonging to a certain GO category, and the color indicates enrichment significance. **c** The distribution of Shannon entropies of transcript ratios of each gene per DGRP gut transcriptome. Uninfected (control) and *P.e.*-infected samples are depicted in gray and brown, respectively. The densities were obtained using R’s base density function. **d** Breakdown of average Shannon entropy per sample by isoform number, susceptibility class, and treatment condition

We used RNA-sequencing data generated from the whole guts of 38 DGRP lines that were infected with *Pseudomonas entomophila* (*P.e.*). Among these 38 lines, respectively 20 and 18 lines are susceptible and resistant to oral infection with *P.e* [13]. In addition, we sequenced the guts of control flies, which were fed sucrose, for a total of 76 samples (Additional file 2). Since the lines are highly polymorphic, we opted to use individualized genomes and gene annotations for our analyses using available single nucleotide polymorphism (SNP), indel, and structural variation data for each line [14] (see the “Methods” section). Given the focus of this study on gaining insights into changes in isoform composition of each gene after infection, we used a multivariate distance-based approach described in [15]. Briefly, we estimated the isoform ratios, that is, the relative ratio of alternative isoforms of each gene, using MISO [16]. We then identified genes showing significant infection-induced differences in isoform ratios [17]. Of the 1877 genes that passed filtering (see the “Methods” section), 40% were significantly changed after infection (Fig. 1a, *p* value of homogeneity > 0.05, BH-corrected *p* value < 0.05, effect size > 0.2, Additional file 3). Interestingly, only 25% of the differentially spliced genes were among the 2471 genes that were differentially expressed after infection, suggesting that gene-level differential expression-type analyses may overlook important molecular aspects of the gut transcriptional response to enteric infection (Additional file 3). Gene ontology analysis revealed that genes associated with mRNA splicing, organelle organization and biogenesis, as well as tissue development are enriched within the set of differentially spliced genes (Fig. 1b). Surprisingly however, this set was not enriched for immunity terms. This may reflect different regulatory properties of genes involved in the immediate innate immune response (i.e., in the resistance mechanisms [20]), many of which are significantly induced after infection, versus those involved in homeostasis (i.e., the tolerance mechanisms [20]), which might be required to function in the normal and infected state. When comparing resistant and susceptible lines within each condition, we were not able to find differentially spliced genes, although some genes showed modest trends (Additional file 1: Figure S1a).

### The transcriptional response is characterized by higher isoform diversity

We next examined the effect of infection on the diversity of the transcriptome by calculating the gene-based Shannon entropy for each sample. This is a measure of the evenness of the proportions of a gene’s isoforms. We found that infection leads to a small but consistent increase in diversity in the infected state (*p* value for treatment effect on average Shannon diversity = 3.7e−05, Fig. 1c, Additional file 1: Figure S1b-c, Additional file 4). The density plot of Shannon entropies revealed that after infection, there is a bias towards increase in the number of genes with higher diversity, and consequently fewer genes with lower diversity, where across the different DGRP strains, there is an average of 20, and a maximum of 330, more genes that increase in diversity after infection (Fig. 1c). This net increase was consistent in 37 different strains irrespective of their resistance class (Additional file 1: Figure S1b), suggesting that this is not a stochastic phenomenon. Interestingly, a breakdown by isoform number revealed that for genes with 2, 3, or 4 isoforms, resistant lines show a tendency for greater average diversity than susceptible lines (Fig. 1d, Additional file 1: Figure S1c). With the exception of genes with four isoforms in the infected state (linear model *p* value for resistance class = 0.0192), this tendency is not statistically significant. These observations suggest that upon infection, the transcriptional output of many genes is less dominated by a single or few isoforms. This phenomenon is more marked in lines that are resistant to *P.e.* infection, which may point to a link between increased isoform diversity and greater infection resistance.

### The effect of natural variation on splicing increases after infection

**Fig. 2:**
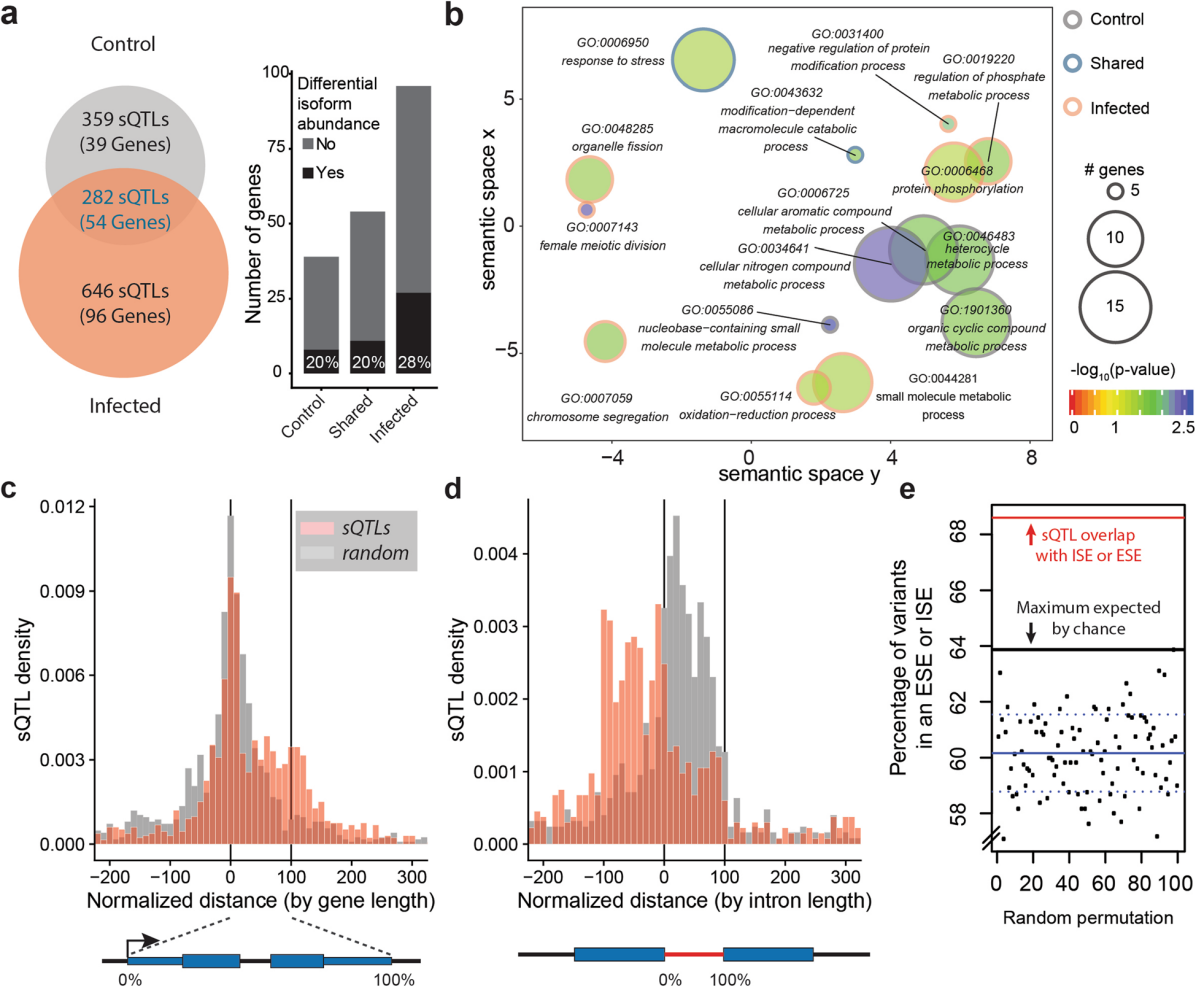
The effect of natural variation on splicing is enhanced by infection. **a** Venn diagram showing the result of the *local-*sQTL analysis (and number of associated genes) using sQTLseekeR [21] (BH adjusted *p* value < 0.05, maximum difference in ratio > 0.1). The barplot shows the number of genes with a *local-*sQTL as well as the overlap with the set of genes with significantly different isoform ratios after infection. **b** GO enrichment of the genes with *local-*sQTLs. The analysis is similar to that in Fig. 1, but the three groups in **a** were tested separately, then the GO categories were pooled in REVIGO. The color of each circle’s outline indicates the gene subset that is enriched with a specific term. **c** Metaplot of the pooled *local-*sQTL results with respect to normalized gene length, and **d** intron length. Orange bars represent the density of *local-*sQTLs, while gray bars represent the density of a random sample of variants that matches the sQTL allele frequencies and is within 10 kb of genes. **e** ESE and ISE locations were predicted along all gene bodies using pattern matching to the reference genome after which the percentage of *local-*sQTLs that overlapped a predicted element was computed and plotted in red. A null distribution of the percentage overlap was produced by randomly sampling variants within gene bodies with a similar allele frequency distribution as the *local-*sQTLs. This was repeated 100 times and the percentage, as well as the mean (blue solid line) and standard deviations (dashed lines) were computed. A solid line shows the maximum overlap obtained through random permutations

We have thus far established that enteric infection leads to a change in isoform abundance of a large set of genes, thereby increasing overall isoform diversity. We next sought to establish whether genetic variation affects isoform ratios. To this end, we identified local splicing quantitative trait loci (*local-*sQTLs) in the two infection states using sQTLseekeR [21]. We restricted our analysis to SNPs within a 10-kb window around each gene (see the “Methods” section), hence our annotation of “*local-*sQTLs”. We identified 359 and 646 control- and infection-specific *local-*sQTLs, and 282 *local-*sQTLs that are common to both conditions (Fig. 2a, Additional file 5). Interestingly, there were around 80% more *local-*sQTLs in the infected state, affecting more than twice as many genes as in the control state (96 vs. 39 genes) although a similar number of genes were tested in the two conditions (1238 vs 1248 for controls and infected, respectively). In addition, a greater percentage of genes with a *local-*sQTL in the infected state showed significant differences in isoform ratios upon infection (Fig. 2a). These results demonstrate that inter-strain differences in isoform ratios can be attributed to alterations in the genomic DNA sequence and that enteric infection unmasks a substantial amount of otherwise silent genetic variants that affect splicing.

To gain insights into which biological processes are enriched in the genes that have *local-*sQTLs, we performed separate gene ontology enrichment of the three sets of genes: control, infected, and shared *local-*sQTLs genes. Figure 2b shows a combined graphical representation of the three GO enrichment results. In the control state, we observed an enrichment of GO terms related to cellular and nitrogen compound metabolic processes. In the infected state, other categories emerged, namely cellular response to stress, cell cycle, and aging. As in the enrichment for infection-induced splicing changes, we did not find any enrichment for immune-related processes, but mostly homeostatic mechanisms. This could either mean that splicing is not a major regulator of canonical immunity pathways or that there is strong selective pressure against genetic variation that affects splicing in immunity-related genes.

Next, we examined the location of the detected *local*-sQTLs in relation to their respective genes. We used a gene-centric and intron-centric approach to obtain metaplots. Since natural variation density along genes is not uniform, and tends to be higher towards the 5′ ends [14], we generated a null distribution by considering sets of randomly selected variants that are located within 10 kb around genes and that have a matching allele frequency spectrum to the *local-*sQTLs. We found that both the null and the observed *local-*sQTL distributions show a peak around the TSS of genes (Fig. 2c, Additional file 1: Figure S2a). However, while the null distribution had a single symmetrical peak with wide tails, the *local-*sQTL density one had a higher density at the main 5′ end, as well as an elevated plateau along the metagene body. This density distribution could be the reflection of multiple possible effects of variants on isoform ratios. One such effect is at the DNA level, where alternative TSS selection could be affected by variants around the 5′ end. Other effects can be through directly modulating splicing all along the transcript. A third type can be modulating transcript stability, which can also be located anywhere on the gene body.

To gain further insights into how *local-*sQTLs could be mediating differences in splicing, we also calculated the *local-*sQTL density distribution around introns as well as a respective null distribution. Interestingly, we observed a pattern that is very distinct from the null distribution. While the latter showed a wide peak that is centered around the 5′ end of introns, the *local-*sQTL distribution exhibited a sharp peak at the 5′ end, with a much greater density of sQTLs immediately upstream compared to downstream of the intron (Fig. 2d, Additional file 1: Figure S2a). In addition, the number of sQTLs dropped sharply at the boundaries of introns. As may have been expected, these data support the notion that genetic variants that affect splicing largely act by inducing differences in the processes that are required for splicing, predominantly around the 5′ splice site. One such *local-*sQTL example is in the gene *Psi*, which has a *local-*sQTL at a splice site (Additional file 1: Figure S2b-d). Lines with different alleles at this locus showed markedly different splicing patterns, with a clear shift in the major isoform produced in both conditions. However, not all *local-*sQTLs could be assigned such a direct mechanism of action, as some might have more subtle effects, for example by affecting exonic and intronic splicing enhancers (ESEs and ISEs) that affect the recruitment of RNA binding factors. To assess this possibility, we asked whether it is more likely that a *local-*sQTL overlaps with an ESE or ISE. Since these splicing enhancer sequences are short hexamers, predicting them along the genome produces many false positives. Nevertheless, we considered a set of 330 published enhancers [22] and looked for matches along all the gene bodies (Additional file 5). We then counted the overlap between the *local-*sQTLs and 100 random sets of variants with a matching allele frequency spectrum. Interestingly, 70% of the *local-*sQTLs overlapped a predicted enhancer, which is 10% higher and 6.1 standard deviations away from the mean of random samples (Fig. 2e). This enrichment indicates that some of the *local-*sQTLs that lie within ESEs and ISEs could be mediating isoform ratios by affecting splicing enhancer function. Taken together, our *local-*sQTL data shows that we can detect effects of natural variation on splicing, even more in the infected state, and suggests that these effects are due to direct changes in splice sites, as well as other mechanisms predominantly at or around the splice donor site. These results also again indicate that splicing changes in the infected state are regulated processes and not merely a result of stochastic perturbations.

### Post-infection transcripts tend to be longer, mainly because of longer 5′UTRs

**Fig. 3:**
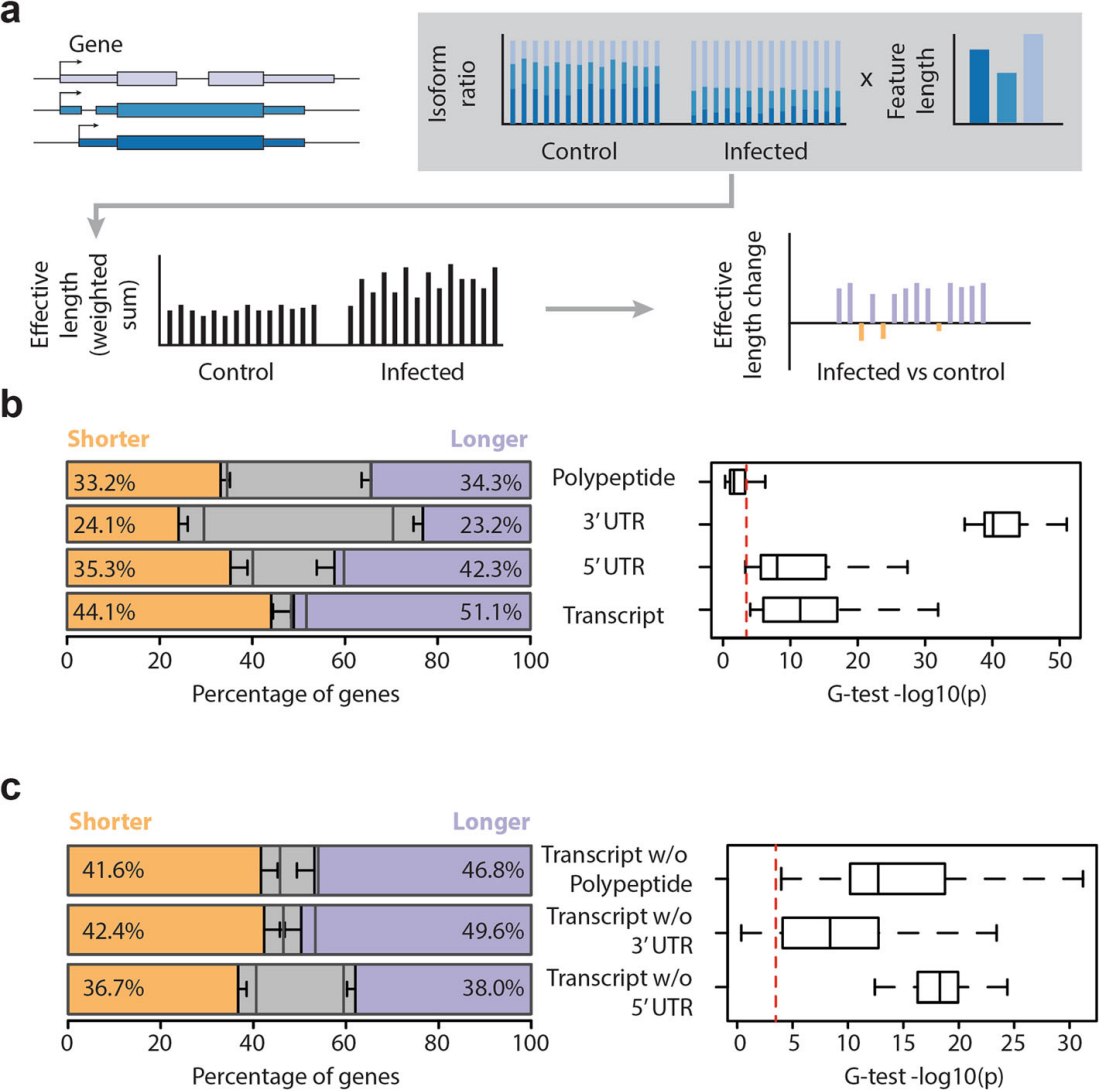
Post-infection transcripts tend to be longer, mainly due to the generation of longer 5′ UTRs. **a** The line-specific effective length of each gene’s transcript, CDS, 5′UTR, and 3′UTR length was obtained by calculating the weighted sum of each gene’s isoform features by its isoform ratios. The difference in effective length between the *P.e.* infected state and the uninfected (control) state was then calculated for each line. **b** The percentage of features that increased, decreased, or did not change in average length (across samples) after infection. Error bars are the standard deviation. A null distribution was generated by performing 100 permutations by randomly shuffling the samples. The gray bars indicate the average obtained by permutations. Repeated G-tests were used to compare the feature length change in each line to the null distribution. The boxplots show the –log_10_(*p values*) of the tests, with the dotted red line representing a Bonferroni-corrected *p value* threshold. **c** Similar to previous panel, but this time the effective length of each transcript without either the predicted polypeptide, 3′UTR, or 5′UTR was calculated

We next sought to characterize the effect of the splicing changes on the length of the produced transcripts. To do so, we estimated an effective length measure for each gene. Briefly, for each gene in each sample, we estimated the effective gene length as the weighted mean of its individual transcripts (taking indels of individual lines into account) by the isoform ratios (Additional file 6). Similarly, we extended this method to specific regions within the transcript, namely the 5′UTR, 3′UTR, and the coding sequence. We then compared the effective length before and after infection to determine the number of genes with an increased, decreased, or unchanged effective length (Fig. 3a). We generated a null distribution of effective length differences by performing 100 permutations of the data, by randomly assigning infection status to the samples, and compared this to our observed set using G-tests. The effect of indels on the coefficient of variation in feature length—that is when we calculate the effect that indels have on the sequence length in the DGRPs without taking expression levels into account—was most prominent in 3′UTRs. However, when we factor in isoform ratios, and calculate the variation in effective lengths, 5′UTRs showed the highest variation (Additional file 1: Figure S3a, Additional file 6). 3′UTR lengths deviated the most from the null distribution, and their infection-induced differences were lower than expected. However, the proportion of those that increased in effective length was close to those that decreased in response to infection (23.2% vs. 24.1 respectively, Fig. 3b, Additional file 1: Figure S3b-c). Furthermore, by classifying genes based on how 3′UTRs may affect their effective length, we found no difference in the contribution of polyadenylation site usage and splicing (Additional file 1: Figure S3d). In contrast to the 3′UTR, we found that around 7% more genes increase rather than decrease in transcript and 5′UTR effective length (paired *t* test *p* values = 1.9e−05 and 1.2e−06 respectively). Predicted polypeptide length, however, did not show differences from the null distribution nor any skew. Importantly, the distribution of this shift in effective length was consistent across the DGRP lines, with transcripts and 5′UTRs having an excess of increased effective lengths, thus supporting that this is a reproducible and genotype-independent phenomenon (Additional file 1: Figure S3b-c). To show which feature contributes to the effective length change the most, we performed a similar analysis, this time calculating the infection-induced change in transcript effective length after the removal of a specific feature. We found that the removal of 5′UTR length and not the predicted polypeptide nor 3′UTR abolished this skew in the proportions (Fig. 3c). Together, these results suggest that infection-induced differences in isoform ratios preferentially affect 5′UTRs and favor the production of isoforms with longer 5′UTRs across genotypes.

### Intron retention increases following infection and its prevalence scales with the degree of pathogenicity

**Fig. 4:**
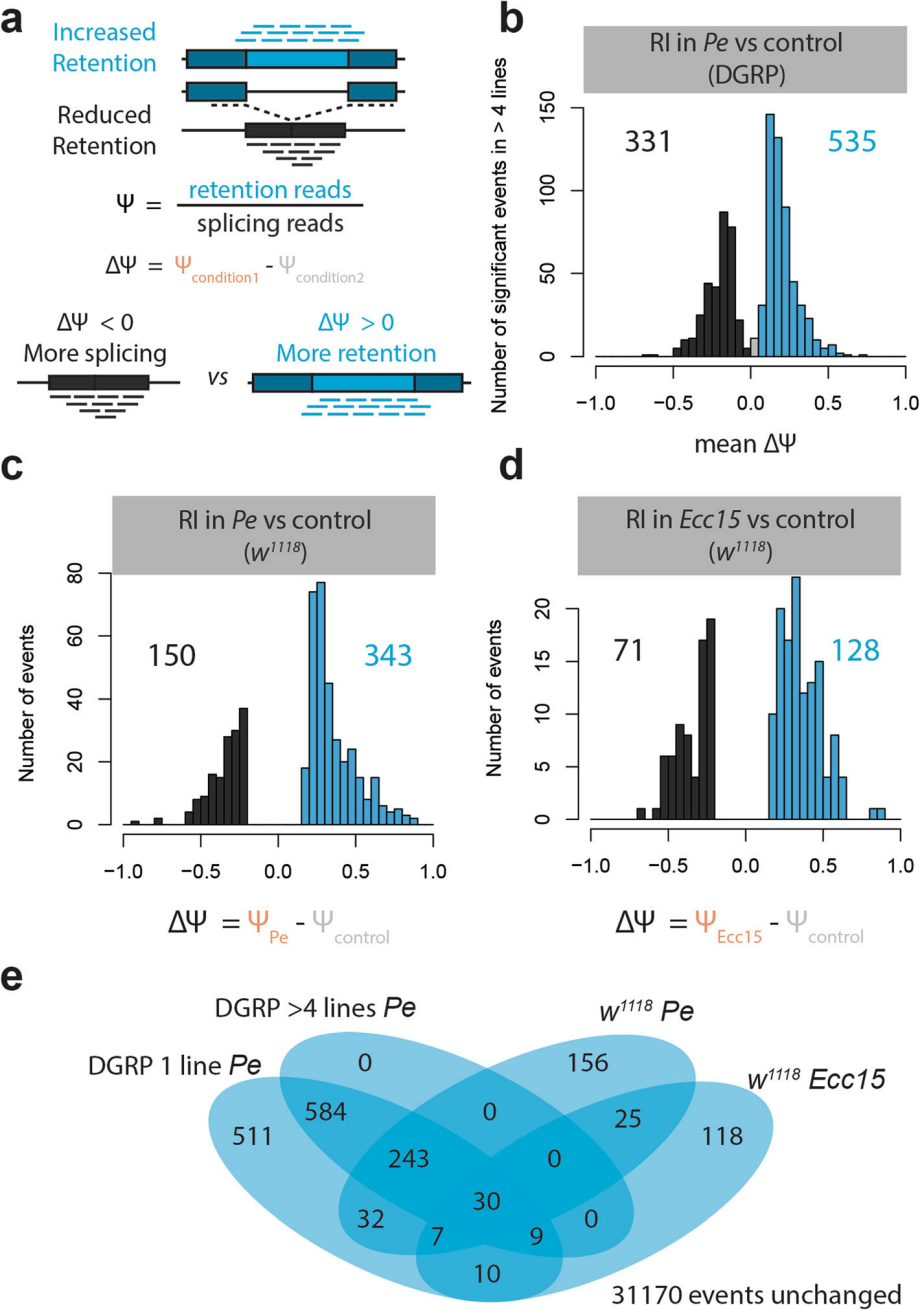
Enteric infection with different pathogens leads to widespread, directed changes in intron retention. **a** Diagram depicting how intron retention changes are calculated. For each sample, delta PSI values for different splicing events [23] were calculated by subtracting the PSI value of the uninfected control sample from that of the infected one. **b** Histogram of average delta PSI values of intron retention (RI) events whose PSI values are significantly different after infection in at least 4 DGRP lines. **c**, **d** Histogram of delta PSI values of intron retention events whose PSI values are significantly different (Bayes factor > 10, delta PSI > 0.2) from the control (sucrose fed) state 4 h after infection with **c** *Pe* and **d** *Ecc15* in *w*^*1118*^ flies. **e** Venn diagram of the overlap between events that are significant in 1 DGRP line, at least 4 DGRP lines, *w*^*1118*^ strain infected with *Pe*, and *w*^*1118*^ strain infected with *Ecc15*

The increase in effective gene length prompted us to investigate splicing at the intron level. Using an available annotation that is specific to intron retention events from the MISO annotation website, we estimated the percent spliced-in (PSI or Ψ) value for each of the 32,895 introns using MISO [16] (Fig. 4a, Additional file 7). This annotation was established based on RNA sequencing of 30 whole animal samples from 27 distinct development stages as part of the modENCODE project [23]. The reliance on two sources of annotations, a gene-centric one with full transcript isoforms from Ensembl and intron-centric one, renders the task of mapping the effect of changes in individual events on whole isoform abundance non-trivial, especially when using short-read sequencing. A limitation that we therefore acknowledge is that not all intron retention events can be directly mapped to an annotated gene. However, despite this limitation, we hypothesized that if a systematic and consistent increase in intron retention based on intron-centric annotations is detected, this may explain why transcripts tend to be longer after infection.

PSI reflects the number of intron retention reads (i.e., spanning the exon-intron boundary as well as the reads in the intron) divided by the sum of the number of intron-retention and intron-splicing reads (i.e., spanning the exon-exon boundary as well as in the flanking exons). In contrast to steady-state analyses, our population-level data from two conditions allowed us to investigate infection-induced changes in intron retention and whether they are restricted to specific transcripts or reflect mere random splicing events. We thereby defined introns with increased retention as introns that significantly increase in PSI (positive delta PSI, bayes factor > 10) whereas introns with reduced intron retention are those that significantly decrease in PSI (negative delta PSI, bayes factor > 10). As shown in Fig. 4b, we uncovered a large number of introns with increased retention (535) and decreased retention (331) that are significant in at least 5 DGRP lines (bayes factor > 10, delta psi > 0.2, also see Additional file 1: Figure S4a-b). These data thus suggest that DGRP strains react similarly to infection. For instance, among the 535 events with increased intron retention in 5 strains, 510 never decreased in retention, 13 decreased in one DGRP strain, 11 in two strains, and one in four strains. Moreover, using the R package SuperExactTest [24], we found that the overlap of introns with increased retention between strains was highly significant. For example, the expected overlap in two and four DGRP lines is less than 10 and 0.001 events, respectively, whereas the median observed overlap was 133 and 59, again suggesting non-random RNA splicing changes. Interestingly, there were 1.6 times more events with a positive compared to a negative delta PSI (535 vs 331 respectively), indicating a net increase in retention post-infection.

It is not clear whether the observed intron retention change is specific to *P.e* and whether different pathogens induce a similar response. We addressed this point by generating paired-end RNA-sequencing data of adult female guts of the widely used *w*^*1118*^ strain infected with the lethal *P.e.* and a non-lethal pathogen, *Erwinia carotovora carotovora 15* (*Ecc15*). Adult female flies were either fed with sucrose (1.5X), *P.e* (OD_600_ = 100 and 1.5X sucrose), or *Ecc15* (OD_600_ = 100 and 1.5X sucrose). When we compared the two infection conditions to the uninfected control state, we found that both conditions differed from the control in intron retention events (Fig. 4c, d, 493 and 200 events in *P.e.* and *Ecc15* respectively, bayes factor > 10, delta psi > 0.2). In addition, we found a high degree of overlap among the DGRP lines, as well as between the DGRP and the *w*^*1118*^ data (Fig. 4e), supporting the notion that this phenomenon deterministically affects a specific set of introns. Nevertheless, *Ecc15* infection yielded fewer differences overall and had proportionally fewer retention events, 40% of which were shared with the *P.e.* condition (Additional file 1: Figure S4c-d). While we only tested infection as an insult in this study, we nevertheless speculate that other interventions may lead to similar changes in splicing. Thus, we postulate that infection-induced splicing differences occur in response to different pathogens, and scale with the degree of virulence, infection severity, or stress.

### Introns with increased retention have exon-like characteristics and are enriched for known RNA-binding motifs

**Fig. 5:**
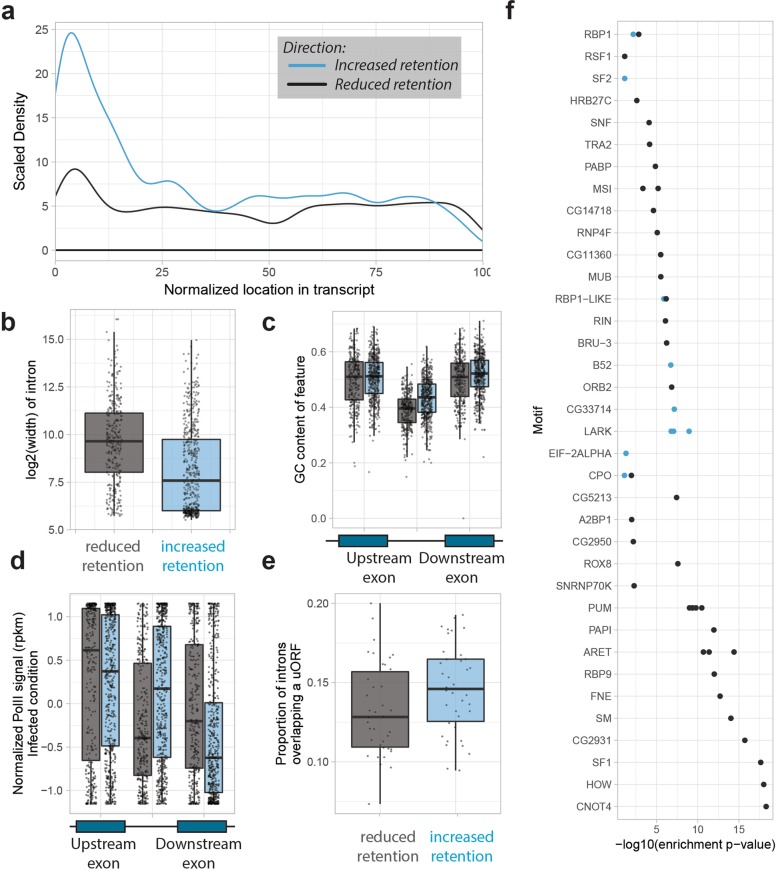
Introns with increased retention have exon-like characteristics. Throughout the figure, blue and gray represent retained and spliced out introns, respectively. **a** The density of intron retention events along the normalized length of the gene. **b** Length of introns (in log_2_) with significant intron retention changes (one-tailed *t* test *p* value < 2.2e−16). **c** GC content of those introns and their flanking exons. **d** Normalized PolII ChIP-seq signal of these introns and their flanking exons in the P.e.-infected state. **e** Proportion of significant intron retention events that overlap with a uORF (paired one-tailed t test *p* value = 8.2e−8). **f** The enrichment of *D. melanogaster* RNA binding motifs [25] calculated using AME [26], in the MEME suite [27]. Blue and gray points indicate enrichment among the sequences of introns with increased and decreased retention, respectively

We next aimed at characterizing the retained and spliced introns. A meta-analysis of the location of introns with increased and decreased retention showed that the density of introns with increased retention is very high at the 5′ end of transcripts, which partly explains why longer UTRs are being produced after infection (Fig. 5a). We then compared their length and GC content, both of which are known parameters that determine exon and intron specification [28, 29]. In terms of length, introns with increased retention tend to be shorter than the ones with decreased retention (Fig. 5b, Additional file 1: Figure S5a). In addition, their GC content tends to be higher, and consequently, the difference in GC content between the introns and their flanking exons was lower (Fig. 5c). Next, we performed RNA Polymerase II ChIP-seq on female guts under control and infected conditions to consider its intron occupancy as an additional characterization parameter (see the “Methods” section). Interestingly, we found that introns with increased retention also show greater enrichment for RNA polymerase II irrespective of treatment condition (Fig. 5d, Additional file 1: Figure S5b, see the “Methods” section). We did not find any enrichment of biological processes for the genes affected by intron retention. Together, these results suggest that retained introns tend to exhibit exon-like characteristics. To formally and independently validate this hypothesis, we overlaid a list of experimentally verified *Drosophila* upstream Open Reading Frames (uORFs) with our data [30]. We found that introns with significantly increased retention in more than 4 DGRP lines are more likely to contain a uORF (paired one-tailed *t* test *p* value = 8.2e−8, Fig. 5e, see the “Methods” section). In fact, when we investigated introns with increased retention in each DGRP line separately, we found that there is generally a greater proportion that overlaps a uORF (Additional file 1: Figure S5c)*.* Thus, our observations suggest that many introns with increased retention may act as uORFs.

The extensive overlap in introns with increased retention among DGRP lines suggests that this process is driven by a deterministic mechanism, possibly involving specific RNA-binding proteins whose differential activity may be responsible for the observed differences. Indeed, it is known that RNA-binding proteins contribute to splicing by binding specific targets in nascent transcripts in a context-dependent manner [31, 32]. We therefore assessed enrichment of RNA-binding motif (RBM) sites in the introns with decreased and increased retention, using as background those introns that did not change significantly. We used AME [26], from the MEME suite [27], to determine enrichment of experimentally derived RBMs in the sequences of introns and the 50 bases flanking them from each side [25]. We found enrichment of many RBMs in the introns with decreased retention, but few RBMs in those with increased retention (Fig. 5f, Additional file 1: Figure S5d,e). Furthermore, upon scanning for motif sequences in these introns, we observed that introns with increased retention not only have more predicted motif binding sites, as expected because of their longer sequences, but also tend to have more motif matches close to the introns’ 5′ splice site. These results suggest that introns with increased retention after infection have generally weaker and fewer splicing signals than those introns that efficiently undergo splicing.

### The RNA-binding protein Lark mediates gut immunocompetence

The lower number of enriched RBMs in the introns with increased retention may indicate that intron retention is generally driven by infection-induced splicing impairments. However, the fact that these introns are shared across inbred lines and distinct pathogens suggests the involvement of a non-random process. To further address this hypothesis, we focused on Lark, since its RBM was the most enriched in the sequences of introns with increased retention, and investigated its possible involvement in the gut’s response to infection. Lark is the ortholog of human RBM4, an RNA-binding protein implicated in splicing, translation, and the stress response. In humans, it has been shown to be activated through phosphorylation by the p38 MAPK pathway in response to stress, where it shuttles out of the nucleus and affects translation of different targets [5]. The MAPK pathway, specifically through *p38c*, has been shown to mediate the *Drosophila* gut immune response to enteric infection through its effect on the transcription factor Atf-2 [33].

**Fig. 6:**
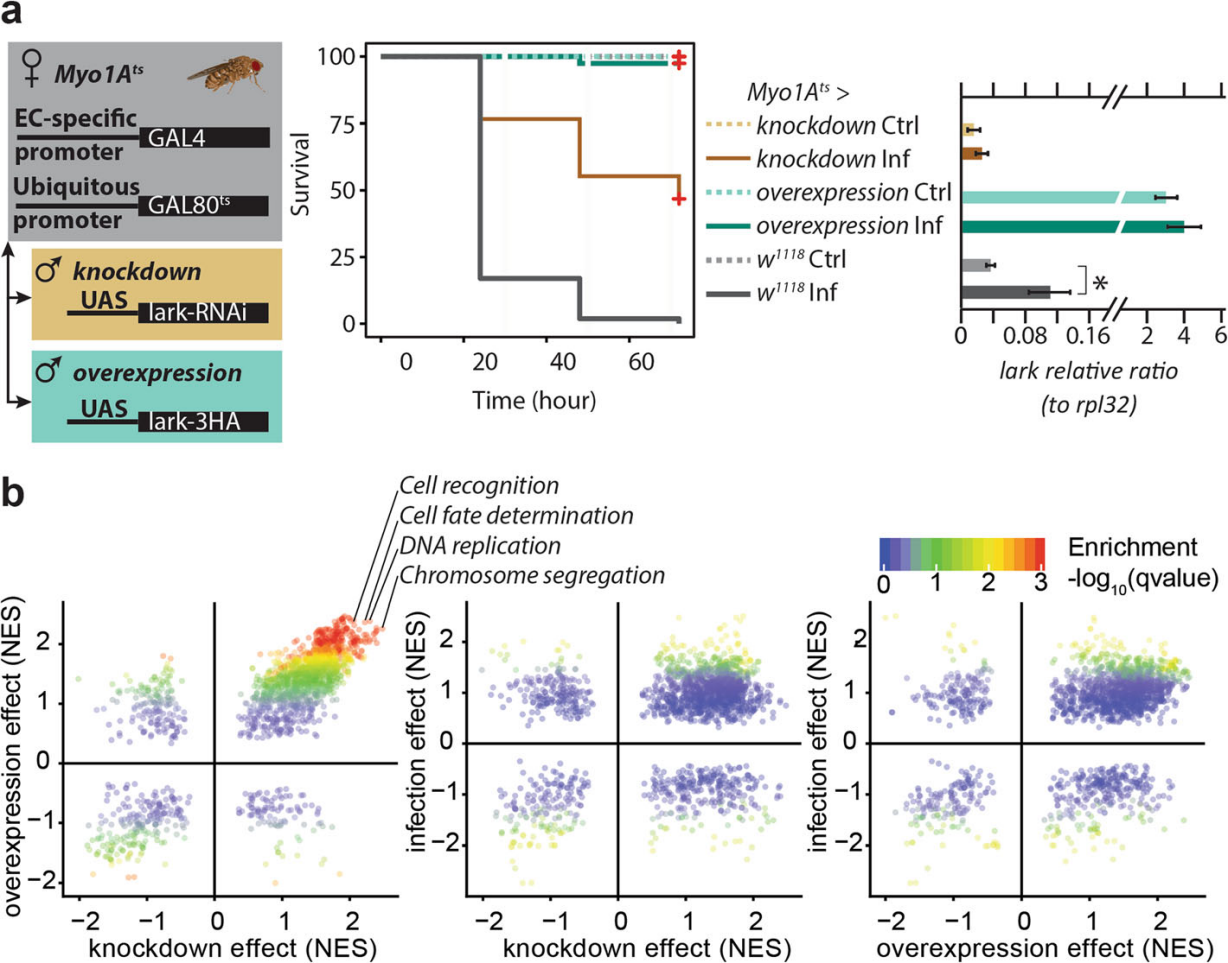
Lark dosage perturbation leads to global changes in gene expression as well as enhanced survival to infection. **a** Left: general schematic of the crosses to generate enterocyte (EC)-specific expression of transgenes in adult female flies. *Myo1A*^*ts*^ virgins were crossed to either *UAS-lark RNAi*, *UAS-lark-3HA*, or *w*^*1118*^ males, and their F1 progeny were maintained at 18 °C. After eclosion, adults were kept at 29 °C for 7 days, then infected with *P.e*. Middle: survival of *lark* overexpression and knockdown flies driven by the *Myo1A*^*ts*^ Gal4 driver. Right: relative ratio of *lark* in dissected guts of those flies 4 h after infection with *P.e*. All experiments were performed with three biological replicates and *n* > 30 flies or guts. **b** Gene set enrichment analysis of the *lark* perturbation effect and infection effect as obtained by gene-level differential expression analysis. Each point is a gene set from the biological process gene ontology whose normalized enrichment score (NES) is plotted in two analyses. Overexpression and knockdown lead to similar changes in gene expression and common pathway enrichments

To investigate Lark’s involvement in the defense response, we performed overexpression and knockdown specifically in the adult gut enterocytes using the *Myo1A-Gal4* driver in conjunction with *tub-Gal80*^*ts*^ (*Myo1A*^*ts*^). Surprisingly, we observed that both knockdown and overexpression of *lark* in adult enterocytes resulted in enhanced survival compared to WT (*Myo1A*^*ts*^ *> w*^*1118*^*)*, with the overexpression transgenic flies being the most resistant to *P.e.* infection (Fig. 6). We validated *lark* knockdown and overexpression by performing RT-qPCR on dissected guts and found that indeed, there was up to 80% knockdown and 80–100 times overexpression in comparison to WT levels. Our observations point to a significant contribution of Lark in the gut response to infection, whereby modulation of its expression levels (either up or down) significantly impacts on overall pathogen susceptibility.

The experiments described above do not provide insights, however, into whether Lark affects intron retention. We therefore performed RNA-sequencing of control and infected guts of flies in which *lark* was overexpressed or knocked-down in adult enterocytes. We first performed gene-based differential expression analysis to characterize Lark-mediated differences. Interestingly, compared to the control and in line with our phenotypic observations, both Lark perturbations led to similar expression differences in terms of genes and gene sets (Fig. 6b, Additional file 1: Figure S6b, Additional file 8). Notably, we observed an enrichment for gene sets related to cell fate determination and cell recognition in the upregulated genes.

**Fig. 7:**
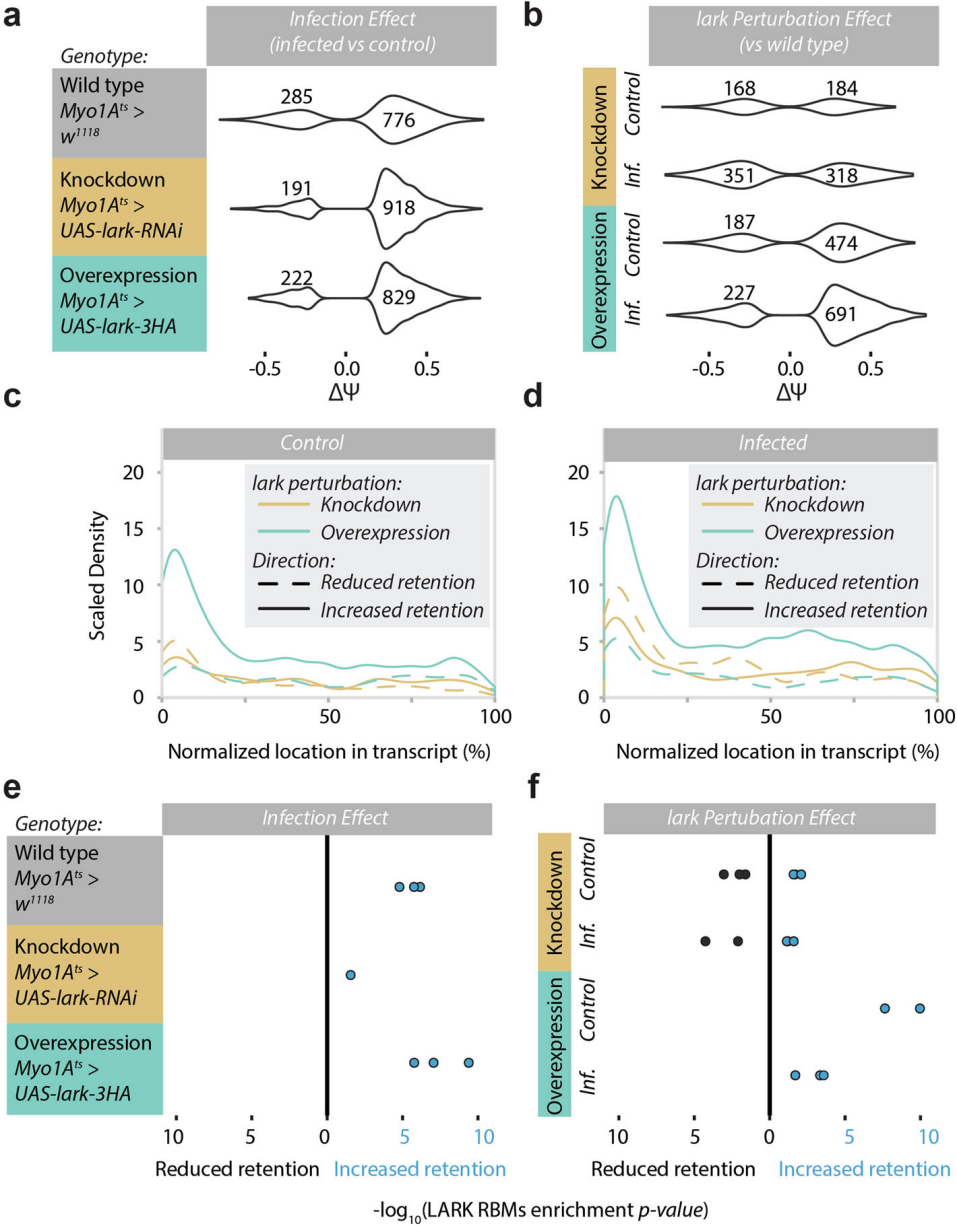
Lark preferentially affects intron retention in the 5′ end of transcripts. **a** The effect of infection on intron retention in the wild type and adult enterocyte-specific knockdown and overexpression of *lark* (using a *Myo1A*^*ts*^ driver in conjunction with UAS-*lark*-RNAi for knockdown and UAS-*lark*-HA for overexpression). Violin plots show the density of delta PSI values of significantly altered events when comparing the *P.e.* infected vs. control samples. **b** The effect of *lark* perturbation on intron retention. For each condition, the knockdown or the overexpression transcriptome is compared to the wild type. **c**, **d** The density of the intron retention events along the normalized length of the gene in the control (**c**) or infected (**d**) condition for each of the *lark* perturbations. **e**, **f** The Lark RBM –log_10_(*p value*) of enrichment in introns with increased and decreased retention compared to non-significantly changed introns. Note that there are three closely related RBMs in the database. The enrichment values of introns with decreased retention are flipped on the *x*-axis for illustrative purposes. **e** The infection effect, and **f** the genotype effect within conditions. Blue and black dots denote Lark RBM enrichment in introns with increased and decreased retention, respectively

We performed the same intron retention analysis as before, but this time, we compared guts with perturbed *lark* expression to wild type (control and infected). We observed a similar increase in intron retention in all the genotypes, meaning that Lark is not strictly required for infection-induced intron retention (776, 918, and 829 events in the control, knockdown, and overexpression flies, Fig. 7a). However, when compared to infected wild-type guts, their *lark* knockdown counterparts exhibited less intron retention (318 vs 691 events, Fig. 7b). Interestingly, overexpression of *lark* led to an important increase in intron retention, even in the control state (474 and 691 in control and infected, respectively, Fig. 7b), and the distribution of introns with increased retention remained concentrated at the 5′UTR, especially when *lark* was overexpressed (Fig. 7c, d). In addition, enrichment of the Lark RBM in introns that were retained due to infection was proportional to *lark* levels (Fig. 7e). Moreover, introns with increased retention due to *lark* overexpression in the uninfected state were also enriched for the Lark RBM (Fig. 7f), indicating that increasing Lark levels directly leads to intron retention of a specific set of genes. We also found an enrichment of the Lark RBM in the introns that are less retained in the knockdown compared to controls (Fig. 7f), providing further evidence for the direct contribution of this RNA-binding protein in infection- and stress-induced splicing regulation.

## Discussion

The gut response to infection involves concerted mechanisms that optimally lead to the clearance of the pathogen and the restoration of cellular homeostasis. An organism must quickly and reversibly adapt to the challenge to ensure survival. Transcription factors that act in response to stimuli, such as Relish (the IMD pathway), Atf-2 (MAPK pathway), and STAT92E (JAK/STAT pathway), have all been studied in the context of gut infection, damage, and regeneration [9, 34–37], and we and others have characterized the regulatory function of those transcription factors [9, 36, 38, 39]. However, another major aspect of transcriptional regulation, splicing, has so far been largely ignored. Here, we present the first characterization of the splicing differences that occur after enteric infection by using a genetic reference panel of flies as well as standard laboratory strains. We found that infection induces widespread and consistent splicing changes in 38 *Drosophila* strains. Only 25% of the genes in our data that exhibited significant infection-induced splicing differences were also differentially expressed after infection. This suggests that splicing is another, so far underappreciated, component in the organism’s defense against enteric pathogens through the generation of molecular diversity. This is in line with our observation of a consistent increase in isoform species diversity, as measured by the Shannon diversity index, after infection. This diversity might be favored in times of cellular stress, expanding the cell’s repertoire of transcriptional products. Intriguingly, this increased diversity is also coupled to an increase in splicing QTLs. The identification of twice as many *local-*sQTLs in the infected state as well as their relative location with respect to genes points to an important role for genetic variation in shaping the gut response to infection. This opens up the possibility that genetic predisposition to stress can be mediated through altered levels of splicing, even though we observed only mild differences in splicing between susceptible and resistant lines. We thereby note that, since our analyses rely on existing annotations of full transcripts, we cannot rule out the possibility that these annotations may be incomplete or that local genetic variation may lead to the expression of novel isoforms. Nevertheless, it is likely that our identified sQTLs on annotated isoform abundance are still valid indicators of genetically driven differences in splicing, yet the exact number of isoforms and the contribution of individual splicing events on the ratios may not be completely accurate. Altogether, we believe that variation in gut immunocompetence cannot be explained by a single pathway or mechanism given the multifaceted and complex nature of this phenotype, but rather by a combination of different factors of which splicing may be another important one [13].

Since we are sequencing poly-A enriched RNA, and not nascent RNA, this diversity increase can possibly be explained by the presence of a mixture of pre-infection and post-infection mRNA species in the same cell and/or by an increased heterogeneity across cells. Other hypotheses can be equally valid. For instance, it could be the result of a general loss of fidelity of transcriptional and post-transcriptional mechanisms, leading to an increase in molecular noise. Studies in human cell lines have shown that noisy splicing is a common feature of genomes [40]. Studies in yeast have shown that gene expression noise confers a fitness advantage after acute severe stress [41], much like our infection model. Indeed, we observed fewer splicing differences and intron retention after *Ecc15* infection, a non-lethal enteric pathogen. However, our findings clearly revealed that infection leads to consistent differences in splicing and intron retention across genotypes and types of pathogens. This observation argues against the increased-noise theory and in favor of a deterministic process that may constitute a more general response to stress, thus beyond the infection model used here.

Our findings on the extent and nature of introns with increased retention are consistent with earlier work showing that widespread intron retention in humans and mouse samples under steady-state conditions is coupled to RNA Pol II pausing [42]. In addition, reduced intron length and higher GC content were revealed as predictors of intron retention [28]. Nascent RNA-sequencing experiments in *Drosophila* S2 cells and whole heads, in steady-state, have shown that intron retention tends to be higher in the first intron [43]. Indeed, many of the infection-induced changes in our analyses are at the 5′ end of transcripts, including the 5′UTRs, which means that infection-induced splicing changes could have more of a regulatory rather than coding function. A large portion of retained introns may thereby function as uORFs given our observation that introns with increased retention have a greater likelihood of overlapping with experimentally mapped uORFs [30] compared to introns with decreased retention. Since uORFs have been shown in *Drosophila* to mostly modulate CDS translation efficiency [30], we speculate that the major regulatory function of such uORFs in introns with infection-induced retention is to negatively affect protein translation initiation by competing for ribosomes. Inhibition of translation is a well-documented aspect of the gut response to pathogenic bacteria, so far shown to be mediated by the activation of the GCN2 kinase and subsequent phosphorylation of eIF2α, which in turn results into limited translation initiation [33, 38]. It is thus conceptually intuitive that the observed intron retention program may act as a complementary process to modulating protein translation in response to infection. This may be especially true for specific gene sets including those coding for splicing factors themselves, which tend to be particularly affected by intron retention across systems and species [44]. Nevertheless, the ultimate consequence of intron retention may well be gene- and context-specific as uORFs have also been shown to promote translation such as is the case for the stress-linked transcription factors ATF4 and ATF5 [45–47]. More integrative and targeted proteome or ribosome profiling studies will be required to inform on these different scenarios.

The observation that introns with increased retention are enriched for the Lark motif led us to investigate the involvement of Lark in the gut defense response. In the fly, this gene has mostly been studied in the context of circadian biology and eye development [48, 49]. In mammals, however, several reports have been published implicating its orthologue, RBM4, in the response to stress through regulation of splicing, transcript stability, and translation control [5, 50]. In this study, we found that enteric infection increased *lark* levels, but surprisingly, both lower and higher levels of *lark*, compared to controls, enhanced infection resistance, implying dosage sensitivity. Nevertheless, modulating *lark* levels alone was sufficient to affect intron retention, especially in Lark RBM-enriched introns, irrespective of infection status. These findings therefore identify Lark as a mediator of both infection-induced splicing differences as well as resistance to infection, but the precise relationship between these two processes warrants further investigation.

## Conclusion

In this study, we were able to implicate Lark in infection-induced splicing differences, as well as resistance to infection, but many questions remain unanswered. Lark seems to be intimately involved in the *Drosophila* gut defense response, yet its downstream effect on the expression of its targets is still uncharacterized. Moreover, the factors controlling *lark* expression and induction in the gut are still unknown. Finally, it is not clear whether the action of Lark is a general stress response or whether its action is adapted to the nature and severity of the stimulus. Answering these questions will increase our knowledge about the functional relevance of splicing in the enteric defense and general cellular stress response.

## Methods

### Fly stocks and infection experiments

DGRP lines were obtained from the Bloomington stock center and reared at room temperature on a standard fly medium. The fly medium recipe that we used is the following: 6.2-g Agar powder (ACROS N. 400400050), 58.8-g Farigel wheat (Westhove N. FMZH1), 58.8-g yeast (Springaline BA10), 100-ml grape juice, 4.9-ml Propionic acid (Sigma N. P1386), 26.5 ml of methyl 4-hydroxybenzoate (VWR N. ALFAA14289.0) solution (400 g/l) in 95% ethanol, and 1-L water. We used *w*^*1118*^ and *yw* flies as wildtype. The UAS-lark RNAi line was obtained from the Transgenic RNAi Project (TRiP.JF02783), and the UAS-lark-3HA line was obtained from Bloomington stock center (stock # 7125). The P-element insertion lines in *lark* were obtained from Bloomington stock center (stock #15287 and #22604). Oral infection was performed using a standard protocol as in [13]. Survival was counted every 24 h.

For specific knockdown or overexpression of *lark* in the adult gut enterocyte, F1 lines carrying a copy of the *MyoIA-Gal4* and *tub-Gal80*^*ts*^ transgenes [51], as well as one copy of either the *UAS-IR* or the *UAS-ORF* was kept at 18 °C for 3 days post-eclosion, and then moved to 29 °C for 8 days to activate the *UAS* transgenes*.* Flies were subsequently infected with *P.e.* using the standard oral infection protocol (OD_600_ nm of 100 and 1.5% sucrose) [13].

### RNA extraction

For the all samples in this study, guts from 30 adult female flies were freshly dissected in PBS after 4 h of treatment. RNA extraction was performed using Trizol Reagent (Invitrogen) using the standard protocol.

### RT-qPCR

cDNA was synthesized from 1 μg total RNA using *SuperScript II* enzyme (Invitrogen). qPCR experiments were performed on a StepOnePlus Real-Time PCR system (Applied Biosystems) using Power SYBR® Green PCR Master Mix (Applied Biosystems). Relative gene expression was calculated after normalization to the control *RpL32* mRNA.

### RNA-seq

#### Library preparation and sequencing

For the *w*^*1118*^ and *Lark* perturbation experiments, paired-end Illumina Truseq libraries were generated and sequenced on an Illumina NextSeq 500 for 75 cycles in the Gene Expression Core Facility at EPFL. As for the 76 DGRP samples, single-end Illumina Truseq libraries were sequenced for 100 cycles on an Illumina HiSeq 2500 at the Genomics Technology Platform of the University of Lausanne. All our samples passed quality control as assessed by FastQC version 0.11.2. For the paired-end samples, we used cutadapt version 1.8 to remove adapter sequences as well as bases with a quality score inferior to 20.

#### Mapping to individualized genomes

For each DGRP line, we generated an individualized fasta genome sequence based on the homozygous variants in the published Freeze 2 DGRP genotypes and the Release 5 reference genome. We also generated individualized gene annotations by applying the offsetGTF tool included in the mmseq package [52] on the Ensembl BDGP5.25 gene annotation. For each sample, RNASeq reads were mapped to the respective genome using the STAR aligner version 2.3.0. Reads for each gene were counted using HTseq-count version 0.5.4p3. For non-DGRP samples, we used the reference genome and gene annotation.

#### Differential expression

Filtering was performed separately for each experiment. For the DGRP lines RNA-seq, genes with more than 5 counts in 38 samples were kept. For the w1118 RNA-seq, genes with more than 5 reads in at least 3 samples were kept. For the lark RNA-seq, genes with more than 10 reads in at least 3 samples were kept. We used limma [53] to perform differential expression analysis, specifically the voom [54] function to estimate counts per million as well as sample weights. To account for intra-strain correlations in the DGRP samples, we used the duplicateCorrelation function with strain as a blocking variable. For the lark experiment, we performed 3 replicates, but realized that two had weak infections as judged by hierarchical clustering (Additional file 1: Figure S6a). Thus, we chose one replicate for the downstream analyses.

#### Transcript ratio estimation and comparisons

We used MISO version 0.5.3 to obtain transcript ratios (PSI values) from each of the individualized genomes and annotations. We used the Ensembl BDGP 5.25 as annotation. We also extracted the assigned counts for each transcript from the MISO outputs. For the detection of genes with significantly altered isoform ratios after infection, we used the rasp package (https://www.isglobal.org/en/web/guest/statistical-software), a distance-based non-parametric multivariate approach as described in [15]. We slightly modified the package script in order to obtain the effect sizes of infection on the isoform ratios of each gene, which are normally calculated but not reported. In order to be kept in the analysis, each isoform must have more than one read assigned to it in 90% of the samples. We used 10,000 permutations to estimate significance followed by Benjamini-Hochberg procedure to control false discovery rate.

#### Intron retention analyses

We used available annotations for intron retention analysis from the Graveley lab [23] to estimate the PSI value of each event in MISO. Then, we used the miso-compare function on each sample pair (treated and control) to detect statistically significant differences due to infection. Events with a Bayes factor greater than 10 and a PSI difference greater than 0.2 were considered significant.

### ChIP-seq

#### RNA polymerase II ChIP-seq

For each condition, 100 *w*^*1118*^ adult female flies were killed by submerging them in liquid nitrogen. Guts were dissected on ice and stored at − 80 °C. On the day of the experiments, guts were homogenized in NE Buffer (15 mM HEPES, 10 mM KCl, 0.1 mM EDTA, 0.5 mM EGTA, 350 mM Sucrose, 0.1% Tween-20, 5 mM MgCl2, 1 mM DTT, 1 mM PMSF, protease inhibitor tablet) supplemented with 1% formaldehyde using a douncer and pestle. After 10 min, crosslinking was quenched by the addition of Glycine for a final concentration of 0.125 M. Samples were cleared by centrifuging for 4 min at 4000 rpm and 4 °C. Samples were washed twice with ice-cold NE buffer and twice with ice-cold RIPA buffer (25 mM Tris-HCl pH 7.6, 150 mM NaCl, 0.5% Na-deoxycholate, 0.5 mM DTT, 0.1% SDS, 1% NP-40, protease inhibitor tablet). Finally, samples were resuspended in 130 μl RIPA buffer and sonicated in Covaris E-220 (30 s, Intensity: 175, Cycles per burst 200, Duty 20%, Water level: 10). Samples were then cleared by centrifugation for 10 min, at 4 °C and max speed. At this point, 1% of the total volume was separated as input and stored at 4 °C; then, the remaining amount was diluted 1:5 in IP Dilution buffer (2.8 ml H2O, 3 μl 10% SDS, 7.2 μl 0.5 M EDTA, 33 μl Triton X-100, 50.1 μl Tris-HCl pH 8.1, 100.2 μl 5 M NaCl). We then added 1 μg of antibody (Abcam ab5408) and incubated the sample overnight at 4 °C on a rotating platform. The next day, the sample was transferred to a tube containing 50 μl of magnetic beads (M-280 Sheep Anti-Mouse IgG) blocked overnight in Beads Blocking Buffer (8.77 ml PBS 1x, 1 ml BSA 1%, 10 μl Triton X-100, 220 μl 45% Fish Gelatin) and the mixture was incubated for 2 h at 4 °C on a magnetic platform. Using magnetic racks, beads were washed once with Low Salt Buffer (20 mM Tris-HCl pH 8.1, 150 mM NaCl, 2 mM EDTA, 0.1% SDS, 1% Triton X-100), twice with High Salt Buffer (20 mM Tris-HCl pH 8.1, 500 mM NaCl, 2 mM EDTA, 0.1% SDS, 1% Triton X-100), LiCl Buffer (10 mM Tris-HCl pH 8.1, 250 mM LiCl, 1 mM EDTA, 1% NP-40, 1% NA-deoxycholate), and TE-NaCl buffer (10 mM Tris-HCl pH 8.0, 1 mM EDTA, 50 mM NaCl). In between each wash, beads were incubated 10 min at 4 °C on a rotating platform. After the last wash, beads are resuspended in 500 μl of Elution Buffer (3.24 mL H2O, 50 μl Tris-HCl pH 7.5 1 M, 10 μl EDTA 0.5 M, 1 mL NaHCO3 0.5 M, 500 μl 10% SDS, 200 μl NaCl 5 M) and the input sample was supplemented with the same amount. From then on, both the input and the IP were treated similarly. We first incubated them at 37 °C for 30 min with 900 rpm shaking in the presence of 7.5 μl RNAse A 20 mg/ml. We then added 10 μl of Proteinase K and incubated the sample at 55 °C overnight. The next day, we added 10 μl of Proteinase K and incubated for 1 h at 45 °C. Samples were then spun down for 5 min at room temperature and 2000 rpm, finally, we used 500 μl of samples as starting material for Qiagen PCR purification kit, following the manufacturer’s instructions. We eluted the IP and the input in 30 μl. We used the Qubit dsDNA HS kit to measure the DNA load.

#### Library preparation

Ten nanograms of DNA was transferred to a low binding tube and completed to 55.5 μl with H2O. We added 3 μl of NEBNext Ultra End Repair/dA-Tailing Module Enzyme mix and 6.5 μl of Reaction buffer and incubated each tube at 20 °C for 30 min, then 65 °C for 30 min. The product of the reaction was purified using the Qiagen MinElute PCR Purification Kit; elution was made in 12.5 μl of Elution Buffer. For each tube, an adapter with a different barcode was selected. We used the DNA Quick ligase kit, using 15 μl of 2× buffer, 1.5 μl of DNA quick ligase, and 1 μl of adapter hybrid primer. Mixture was incubated at 22 °C for 30 min. The reaction was purified using the Qiagen MinElute PCR Purification Kit; elution was made in 50 μl of Elution Buffer. Samples were purified using AMPure beads in a 1:1 ratio, washed twice with 80% EtOH and resuspended in 20 μl of Elution Buffer. Using 1 μl, we perform a qPCR using the KAPA SYBR green kit 50 μl total volume to determine the number of cycle for each samples. We then amplify each sample by PCR using the KAPA master mix. We then perform a size selection using AMPure beads, first using a 0.6:1 ratio and excluding the bound fraction followed by a 1:1 ratio selection, washing twice with 80% EtOH and resuspending in 20 μl Elution Buffer. We used in 1 μl to measure the DNA load with Qubit dsDNA HS assay and 1 μl to assess the fragment profile using the Agilent Bio-analyzer DNA 12000 kit.

#### Mapping and analysis

Chip-Seq samples were sequenced on an Illumina Hiseq 2500. The sequencing reads were mapped to the reference genome using Bowtie2 (--end-to-end --very-sensitive); then, the counts for every intron retention event (the flanking exons as well as the intron) were counted using the regionCounts function in the R csaw package [55]. The count data was converted to RPKM and quantile normalized prior to the analyses. Since the RNA pol II coverage decays from the 5′ to the 3′ end of a gene, we converted the RPKM values to the standard normal distribution for each intron retention event (the flanking exons and intron) when we were comparing the retained and the spliced events.

### Statistical and computational analyses

#### Shannon diversity

For each gene, the Shannon diversity was calculated based on the transcript ratios of its annotated isoforms using the Vegan R package [56]. This was done for each RNA-seq sample. The Delta Shannon for each DGRP line was calculated by subtracting the control Shannon diversity from the infected one.

#### Effective length calculations

We first generated tables of transcript, 5′UTR, 3′UTR, and CDS lengths for each line, considering the insertions and deletions in those lines. Then, for each line and condition, we calculated the effective length of a gene as the sum of the products of the length and the corresponding isoform ratio (Fig. 3). To address whether 3′UTR effective length changes were due to differential polyadenylation site use versus splicing, we devised a strategy to classify multi-isoform genes into these two groups. We used simple rules to decide whether 3′UTRs of that gene (1) can contribute to diversity in the first place, (2) have the same number of exons, and (3) share a common start position and different end position. Using this strategy, we obtained three groups of genes (out of a total of 3733 genes with more than one isoform). Splicing: Genes for which splicing can affect the 3′UTR length (*n* = 387). Alternate3Poly: Genes for which an alternate choice of polyadenylation site can affect the length of the 3′UTR (*n* = 1138). No annotated diff/Ambiguous: Genes with either no difference in isoform length or ambiguous classification (*n* = 2208). We then broke down the 3′UTR effective length changes as in Fig. 3b by UTR class. For example, if a gene has more than one isoform, we would first check if it has diversity in 3′UTR lengths of the different isoforms. Then, we would look at those 3′UTRs and check if they are encoded by the same number of exons. If not, then effective length of these 3′UTRs would likely be affected by splicing. If all the transcripts’ 3′UTRs have the same number of exons, and these exons share the same start position but different end position, we put the gene in the “Alternate3Poly” category.

#### sQTL analysis

sQTL analysis was performed using sQTLSeekR [21] using the transcript ratios and genetic variants 10 kb around each expressed gene with multiple isoforms. We performed slight modifications on the package script in order to extract information about the effect size of sQTLs which was normally calculated but not reported.

#### ESE and ISE analyses

We used a published set of 330 intronic and exonic splicing enhancers and pattern matching through the BSgenome and Biostrings R packages to catalogue all the possible locations of those elements within the gene bodies of the reference genome. We then calculated the percentage of sQTLs that overlap with a predicted element. To assess the overlap expected by chance, we randomly sampled, 100 times, sets of variants that are within 10 kb of expressed genes that have a similar allele frequency spectrum as the sQTLs.

#### RNA-binding motif analyses

We used AME version 4.11.1, from the MEME suite, to perform enrichment of all binding motifs of RNA binding proteins using *Drosophila*-specific PWM scores from [25] in introns with increased and decreased retention. The same RNA-binding protein can have multiple RBMs. We used FIMO, also from the MEME suite, for motif scanning using the same set of PWMs. Given that the retained introns were poorly enriched for RBMs and in order to visualize the locations of motifs in both the introns with increased and decreased retention, we used a high FDR threshold of 40% to filter the resulting matches and only kept the motifs that are enriched in the AME results (Additional file 1: Figure S5d,e). For both AME and FIMO analyses, we used the sequences of introns that do not change significantly, that is, introns that are neither spliced nor retained after infection, as background.

#### Overlap with uORF

We used supplementary data table 2 from the study of Zhang and colleagues [30]. We converted coordinates from R6 to R5 using the Flybase Coordinates Back-Converter. We only kept uORFs that are less than 201 bp in length which left us with 32,924 out of 37,619. We looked for any overlap between introns and the uORFs based on the reference locations; then for each DGRP line, we split the intron events by the sign of their PSI value and counted the proportion of those events that have a uORF. To test for significance, we performed a paired one-tailed *t* test between the positive and negative logit-transformed proportions.

## Supplementary information

**Additional file 1: Figure S1.** Enteric infection leads to extensive changes in transcript isoform ratios and increased diversity. **Figure S2.** Example of the functional relevance of a *local-*sQTL. **Figure S3.** Post-infection transcripts tend to be longer, mainly due to the production of longer 5′ UTRs. **Figure S4.** Enteric infection with different pathogens leads to widespread changes in intron retention. **Figure S5.** Introns with increased retention have exon-like characteristics. **Figure S6.** Lark perturbation leads to global changes in gene expression as well as enhanced survival to infection.

**Additional file 2.** RNA-samples used in the study and metadata. Sheet1. DGRP RNA samples used in the study. Sheet2. *w*^*1118*^ RNA samples used in the study. Sheet3. *lark* perturbation samples used in the study.

**Additional file 3.** Infection-induced transcript isoform changes. Sheet1. All PSI values per transcript as calculated using MISO. RASP results for infection effect. GO results for infection effect. Differential expression results for infection effect (companion study).

**Additional file 4.** Shannon diversity results. Sheet1: Shannon diversity per gene per DGRP sample. Sheet2: Delta Shannon diversity (Infected – Control) per gene per DGRP line.

**Additional file 5.** sQTL results. Sheet1. All sQTL results in both conditions (Control and Treated). Sheet2. Significant sQTL results in both conditions, along with predicted coding consequence, location within transcript, and matches to the ESE and ISE. Reference list for ESE and ISI.

**Additional file 6.** Lengths of transcripts and effective lengths of genes. Sheet1. Lengths of transcripts and features from reference annotation. Sheet2. transcript lengths by DGRP line (taking indels into account). Sheet3. CDS lengths by DGRP line (taking indels into account). Sheet 4. 5’UTR lengths by DGRP line (taking indels into account). Sheet5. 3’UTR lengths by DGRP line (taking indels into account). Sheet6. Gene effective length by transcript (taking indels and isoform ratios into account). Sheet7. Gene effective length by 5’UTR lengths (taking indels and isoform ratios into account). Sheet8. Gene effective length by 3’UTR lengths (taking indels and isoform ratios into account).

**Additional file 7.** Intron retention results. Sheet1. Intron retention annotations and results for the DGRP lines. Sheet2. DGRP PSI table per sample. Sheet3. DGRP delta PSI table per strain. Sheet4. DGRP bayes factor table for infection effect comparisons. Sheet5. Significantly changed intron retentions in the *w1118* background (*Ecc15* or *Pe* infection). Sheet6. Intron retention events in the different *lark* perturbation experiments. TRUE means that the event is significantly different in the performed comparison. Sheet7. Summary of overlap with uORF by DGRP line: overlaps were calculated for all, or the significant subset of introns per DGRP strain.

**Additional file 8.*** lark *differential expression and GSEA analyses. Sheet1. Combined differential expression top tables for the three analyses (rnai = RNAi vs WT, uas = UAS.lark vs WT, infection = all infected vs all uninfected). Sheet2. Results of the GSEA analysis based on the logFC values of each gene and gene ontology terms acquired from go2msig.org (April 2015).

**Additional file 9.** Review history.

## Data Availability

The RNA-seq and ChIP-seq datasets supporting the conclusions of this article are available in the NCBI GEO repository, GSE118622 [57]. Analysis scripts are available on Zenodo [58].
