# Supplementary material for: Enteric infection induces Lark-mediated intron retention at the 5′ end of Drosophila genes
Source: Genome Biol. 2020 Jan 17;21:4. doi: 10.1186/s13059-019-1918-6 (PMC6966827; doi:10.1186/s13059-019-1918-6)
Supplement: Supplementary file 1 — Additional file 1: Figure S1. Enteric infection leads to extensive changes in transcript isoform ratios and increased diversity. Figure S2. Example of the functional relevance of a local-sQTL. Figure S3. Post-infection transcripts tend to be longer, mainly due to the production of longer 5′ UTRs. Figure S4. Enteric infection with different pathogens leads to widespread changes in intron retention. Figure S5. Introns with increased retention have exon-like characteristics. Figure S6. Lark perturbation leads to global changes in gene expression as well as enhanced survival to infection. [file 13059_2019_1918_MOESM1_ESM.docx]

**Additional File 1: Supplementary figures for**

**Enteric infection induces Lark-mediated intron retention**

**at the 5’ end of *Drosophila* genes**

Maroun S. Bou Sleiman^1^, Michael Frochaux^2^, Tommaso Andreani^3^, Dani Osman^4^, Roderic Guigo^5^, Bart Deplancke^6+^

## Supplementary Figures


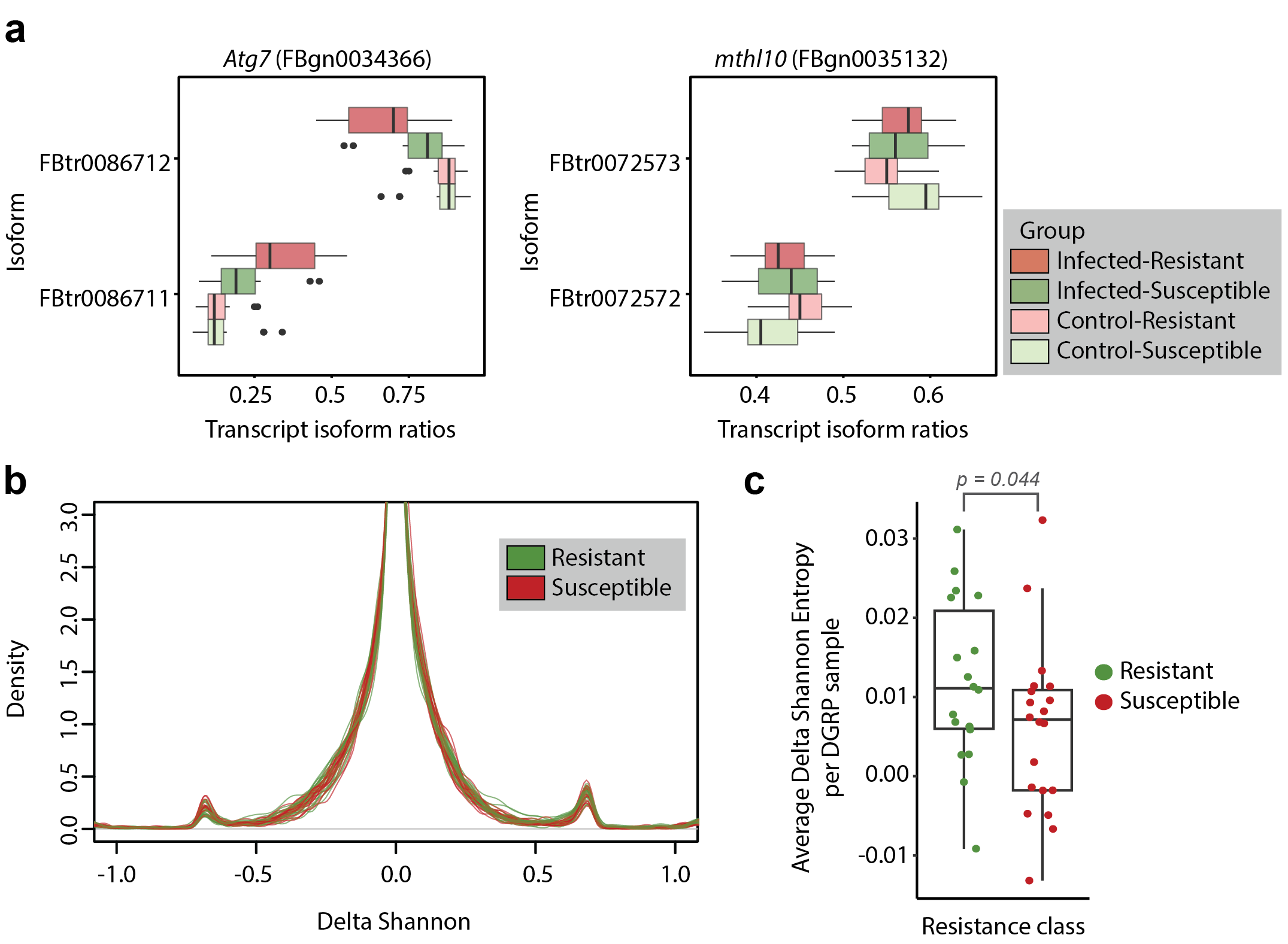


**Fig S1. Enteric infection leads to extensive changes in transcript isoform ratios and increased diversity. (a)** Examples of gene isoform ratios in the different conditions and susceptibility groups. The annotated isoforms of each gene are on the y-axis whereas the isoform ratio is plotted on the x-axis. *Atg7* shows a shift in isoform usage upon infection, whereas *mthl10* exhibits a slight difference in isoform usage between resistant and susceptible lines in the uninfected state. Both genes have two annotated isoforms. **(b)** Distribution of delta Shannon entropy values (Shannon entropy in infected minus uninfected state) per gene per DGRP line. **(c)** Boxplot of average delta Shannon entropy per DGRP line, separated by resistance class (one-tailed t-test p-value < 0.05).


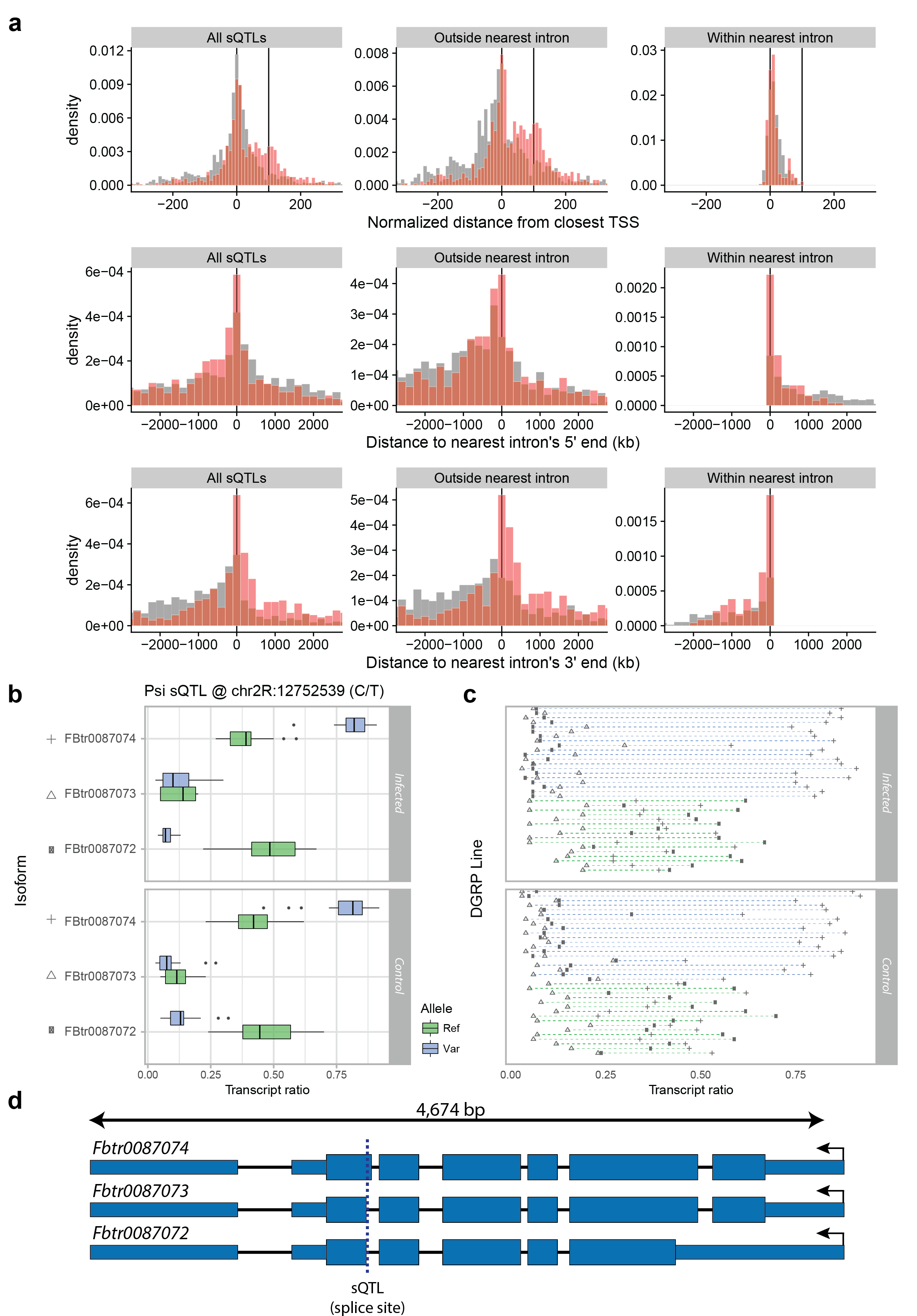


**Fig S2. Example of the functional relevance of a *local-*sQTL.** **(a)** Breakdown of sQTL positions by location and relative to the nearest TSS, 5’ intron splice site, or 3’ intron splice site. sQTLs were further divided into two groups: those that are outside the nearest intron, and those that lie inside the nearest intron. Red bars represent the densities of sQTLs whereas grey bars represent the density of random variants. In the upper panels, the two black vertical lines represent the normalized gene length (from 0 to 100%). **(b)** The isoform ratios of a gene (*Psi*) that has a *local-*sQTL on one of its splice sites. The expression levels are grouped by allele of the *local-*sQTL, with 0,1,2 being reference and alternate alleles, respectively. **(c)** The isoform ratios by DGRP line in the two conditions. The shape of the point indicates the isoform and the color of the dashed line indicates genotype. **(d)** Gene diagram of *Psi* showing the location of the *local*-sQTL.


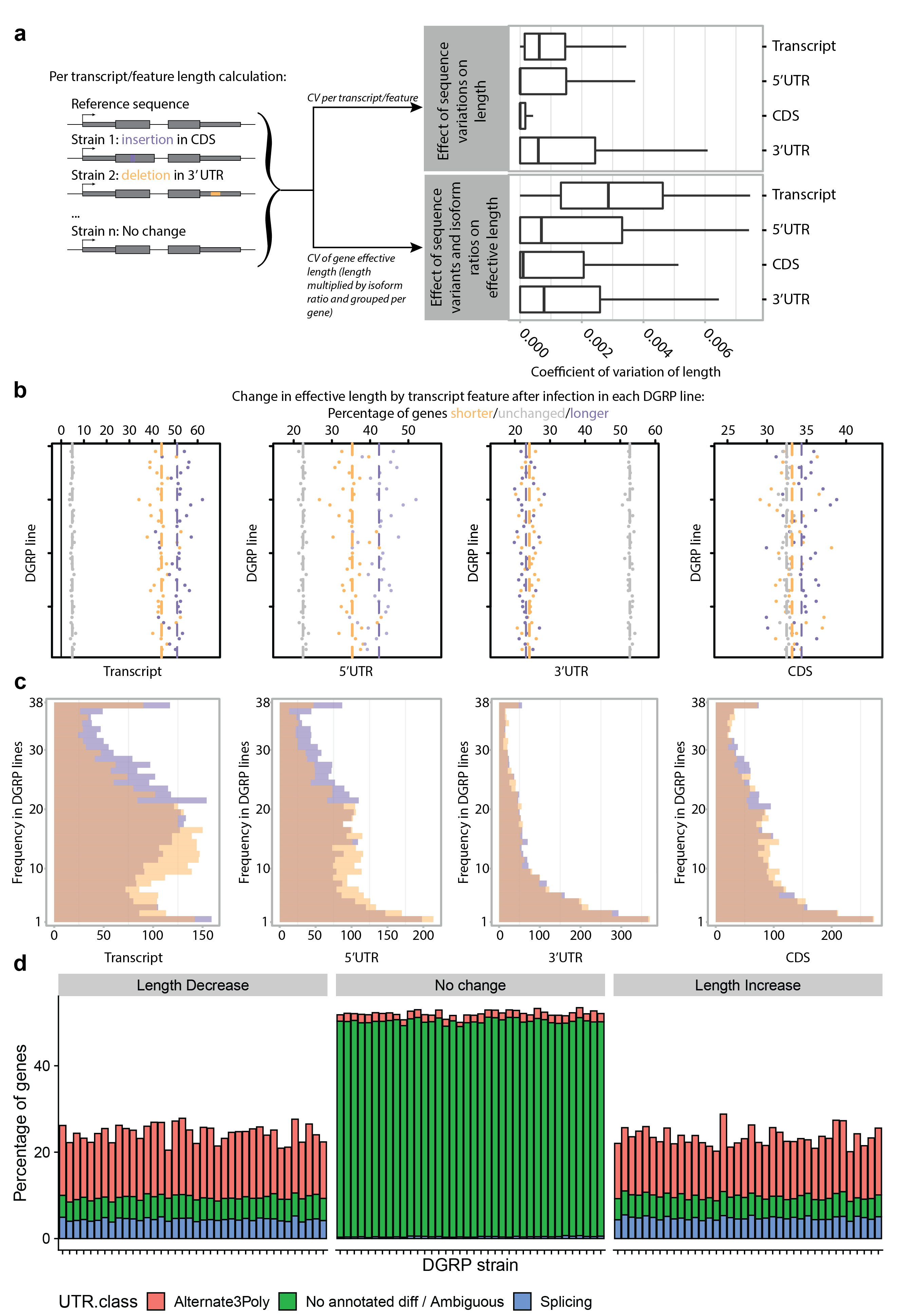


**Fig S3. Post-infection transcripts tend to be longer, mainly due to the production of longer 5’ UTRs.** **(a)** Left panel: The distribution of coefficients of variation in length of each transcript or feature due to natural variation within the DGRP lines. Right panel: The distribution of coefficients of variation in effective length of each gene due to natural variation within the DGRP lines, isoform transcript ratios, and infection. **(b)** Breakdown of number of genes whose effective length increases (in green), decreases (in red) or stays constant (in grey) based on a certain feature (From left to right: the predicted polypeptide- (cds), 3’ UTR-, 5’ UTR-, and the transcript-based effective length change). **(c)** The per-gene frequency distribution among the DGRP lines of the number of genes that increase (green) or decrease (red) in effective length based on the features (from left to right: 3’ UTR, transcript, 5’ UTR, and predicted polypeptide). **(d)** Breakdown of 3’ UTR effective length changes by 3’ UTR class. Each gene with more than 1 isoform, was classified into 3 groups, depending on how its isoform lengths may be affected by 3’ UTRs. Alternate3Poly is the group of genes whose 3’ UTRs vary in 3’ polyadenylation site usage. Splicing is the group that is affected by alternative splicing. The remaining genes are either complex or ambiguous, or do not have diverse 3’ UTRs. The percentage is the percentage of all genes that increase, decrease, or do not change in effective 3’ UTR length.


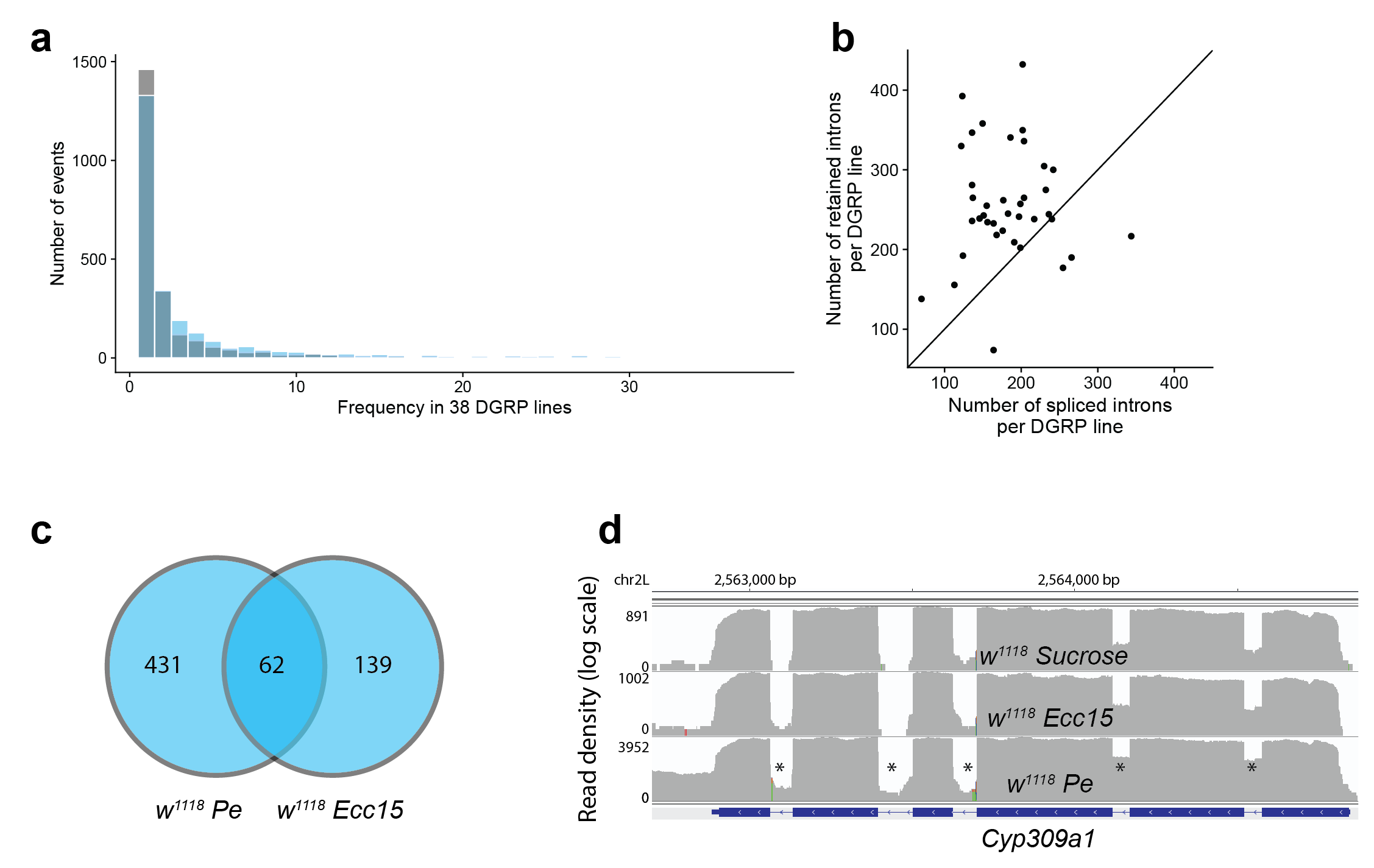


**Fig S4. Enteric infection with different pathogens leads to widespread changes in intron retention. (a)** Frequency distribution of introns with significant increase or decrease in retention in the 38 DGRP lines. The two distributions are overlaid, not stacked, and show that there are more introns with increased retention that are shared between different DGRP lines. **(b)** Scatterplot showing the number of introns with significantly decreased and increased retention in each DGRP line. Most DGRP lines have more introns with increased than decreased retention. **(c)** Venn diagram showing the intersection of the significant intron retention events under the two conditions (*Pe* and *Ecc15*). **(d)** Illustration of multiple intron retention events within a single gene *Cyp309a1*. Retained introns are marked by an asterisk. The snapshot was obtained using IGV.


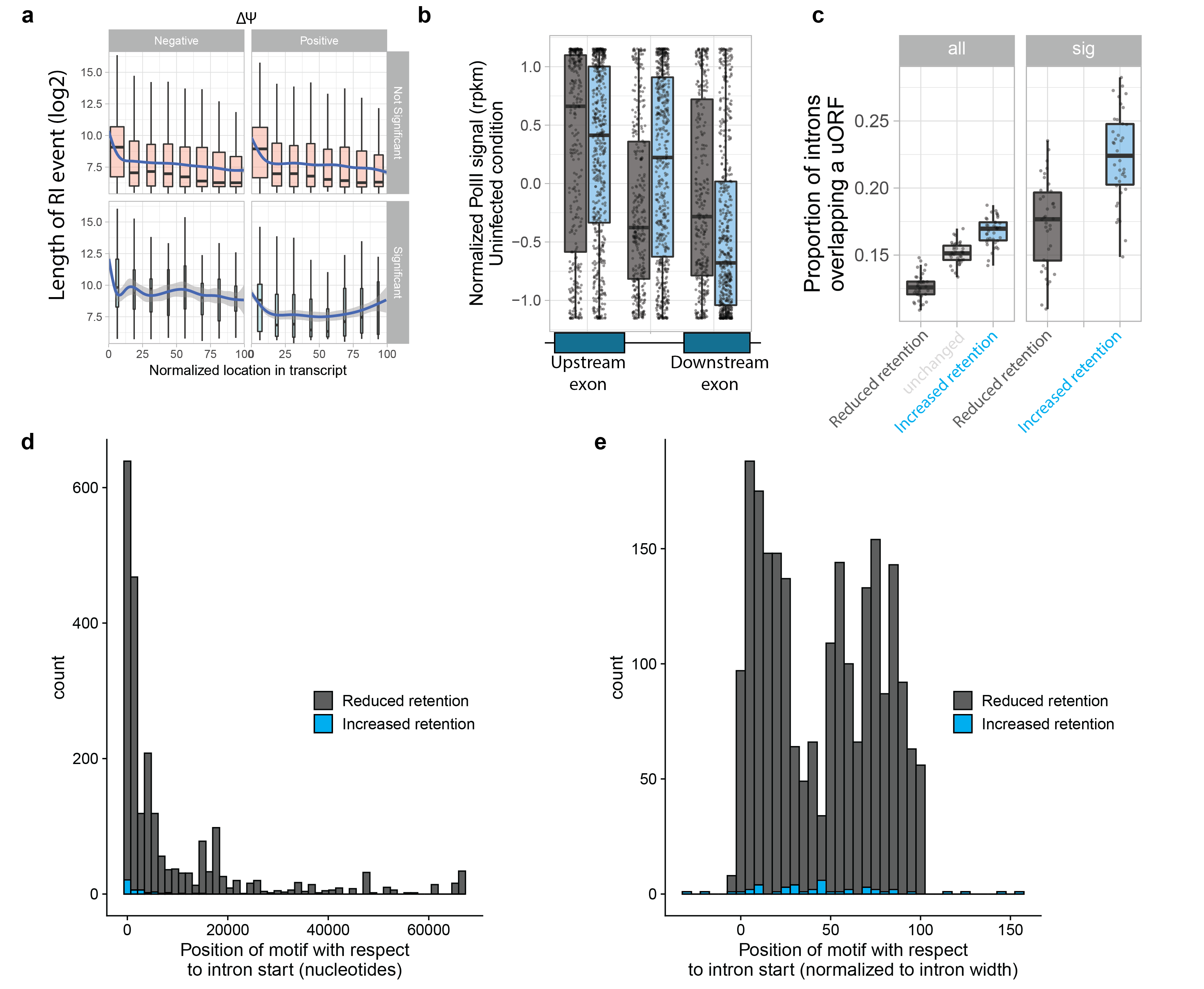


**Fig S5. Introns with increased retention have exon-like characteristics**. **(a)** Distribution of intron lengths (in log_2_ scale) as a function of location within the transcript for non-significant (upper panels, red) and significantly changed introns in at least 4 DGRP lines (lower panels, blue). The left panels show introns that have decreased retention (negative delta PSI) whereas the right panels have increased retention (positive delta PSI, right). The blue lines are loess smoothing curves with 95% confidence intervals. **(b)** Standardized RNA polymerase II signal density in log_2_(rpkm) in the uninfected state, in introns and flanking exons for all intron retention events with significant changes in retention. **(c)** Proportion of introns overlapping a uORF in all tested introns by DGRP line (left panel, paired one-sided t-test p-value = 1.47e-15), and in introns that are significantly changed in each of the DGRP lines (right panel, paired one-sided t-test p-value = 8.2e-8). **(d-e)** Histogram representing the motif scanning results, using FIMO, of enriched motifs in the two intron groups: **(d)** shows the positional density in nucleotides with respect to the introns’ 5’ splice site, whereas **(e)** shows the positional density normalized to the intron length. The scans were performed on introns plus 50 bps from each side, hence some predicted binding sites are found outside the normalized 0-100% range of the intron.


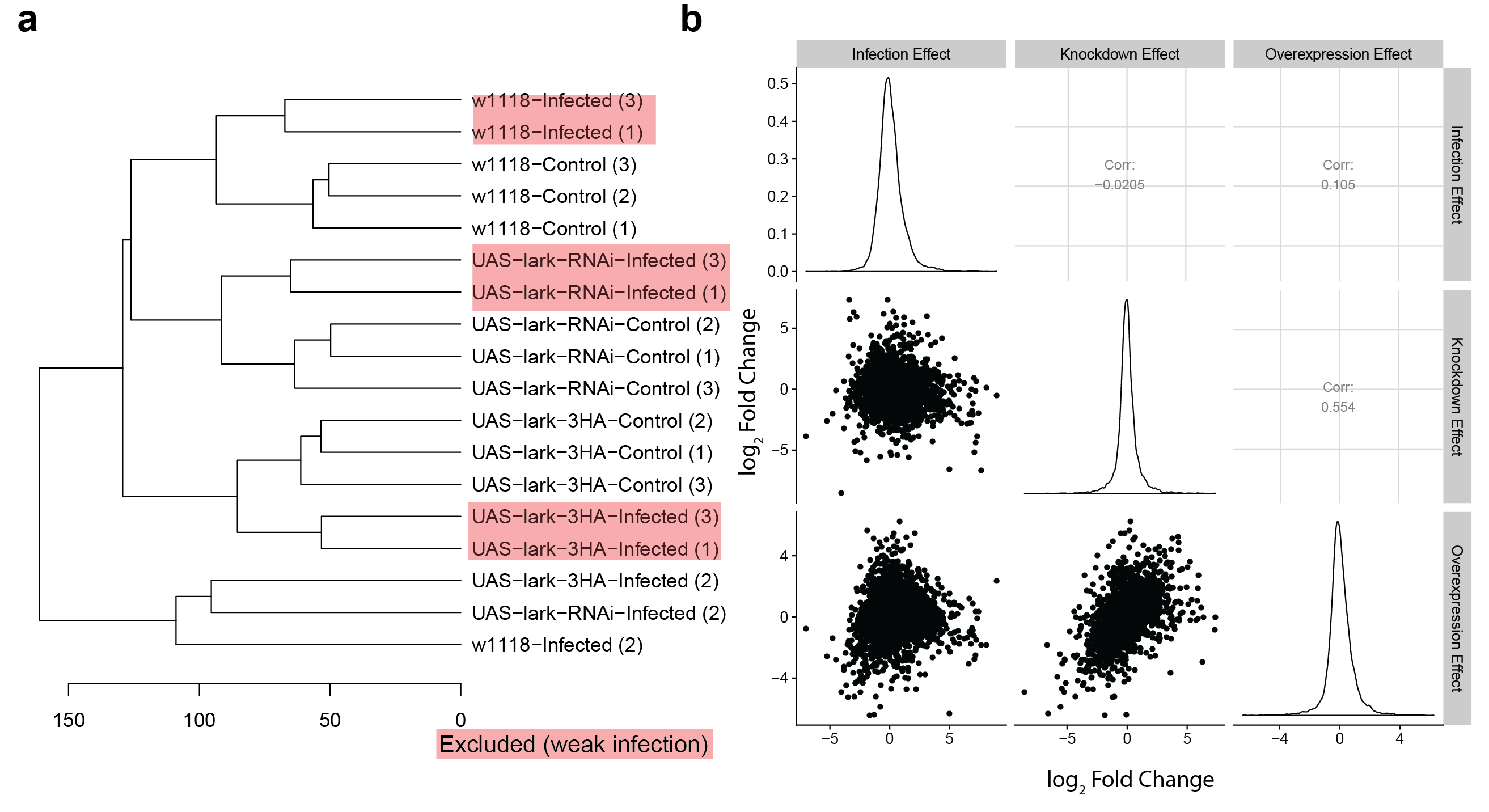


**Fig S6. Lark perturbation leads to global changes in gene expression as well as enhanced survival to infection. (a)** Hierarchical clustering of the generated transcriptomes. We identified two batches where the infection effect was weaker than the genotype effect, so we excluded them from further analyses. **(b)** Pairwise comparisons of log_2_(fold changes) of the infection, overexpression, and knockdown effects. The fold changes were computed using limma with a model that includes genotype (WT, *lark* knockdown, *lark* overexpression) and condition (control and infected).
