# Supplementary material for: Enteric infection induces Lark-mediated intron retention at the 5′ end of Drosophila genes
Source: Genome Biol. 2020 Jan 17;21:4. doi: 10.1186/s13059-019-1918-6 (PMC6966827; doi:10.1186/s13059-019-1918-6)
Supplement: Supplementary file 9 — Additional file 9. Review history. [file 13059_2019_1918_MOESM9_ESM.docx]

Review History

**First round of review**

**Reviewer 1**

**Are you able to assess all statistics in the manuscript, including the appropriateness of statistical tests used?** Yes, and I have assessed the statistics in my report.

**Comments to author:**

This manuscript looks at the changes to splicing that occur during the Drosophila gut immune response. This is to my knowledge entirely novel - there have been numerous studies on transcriptional responses to infection but these have tended to be restricted to transcript abundance. The results are striking, with extensive intron retention extending the length of 5'UTRs. The authors then convincingly link this to an RNA binding protein called Lark. Knocking-down or overexpressing this protein affects susceptibility to infection, suggesting this may be important in resisting infection. I found this to be an important advance in our understanding to the response to infection and splicing more generally.

The manuscript leaves open the critical question of the function of intron retention in the 5' UTR. However, this is sensibly discussed and I am content this falls beyond the scope of this manuscript. I was also left wondering whether this was a general stress response or a more specific immune response, but again feel this can be left for the future. Infection in flies can cause quite dramatic changes to the gut tissues, so different infection regimes may alter the abundance of different cell types in the samples. It would be nice to have a few words describing how guts respond to this particular infection and the consequences of this for the reults. In places the inclusion of natural genetic variation felt peripheral to the main narrative.

The concise description means it would be challenging to reproduce the analysis. Making scripts public can solve this.

**Reviewer 2**

**Are you able to assess all statistics in the manuscript, including the appropriateness of statistical tests used?** Yes, and I have assessed the statistics in my report.

**Comments to author:**

In this paper, the authors have examined the effect of enteric infection on gene expression, including differential alternative splicing, in the Drosophila Genetic Reference Panel inbred lines. They find that while there are few differences in alternative splicing between resistant and susceptible lines, infection has dramatic effects on alternative splicing and reveals cryptic genetic variation in splicing (infection-dependent sQTLs). On average, post-infection transcripts tend to be longer, mainly due to 5' UTRs.  These results are significant, and will interest anyone concerned about immunity or the regulation of splicing.

However, "extensive intron retention" (in the title) focuses excessively on one aspect of the observed phenomenon and is an overstatement.

There are many analyses here, and some are poorly described. The most important of these concerns the "spliced introns" comparison set. The identification of lark as a factor of interest is based on a comparison of retained and "spliced" introns (Fig. 5f).  I understand that "retained introns" is used here to refer to introns that are preferentially retained in response to infection. But what are the "spliced" introns? This set is used many places in the paper but never defined.  All introns are, by definition, spliced, so the term itself is not useful. The number of "spliced" introns and their properties also suggest that a specific definition is being used. Methods (ll. 599-600) states that introns that do not change significantly after infection were used as background in the RNA-binding motif analyses, so "spliced introns" must be a subset. One guess is that the "spliced introns" are those that do change significantly, but which are not retained intron events. These "spliced introns" would therefore be infection-dependent changes other than intron retention (i.e. alternative 5' splice site, cassette exons, etc.).  Another possibility is that the "spliced introns" are those whose retention goes down following infection. This seems unlikely since this class would also be retained introns, and the same intron could be in different categories in different lines.  

Fig. 5f shows that the RNA-binding motif enrichment in set of "spliced introns" is, in general, much greater. We should be told more about this set of introns and the location of these more-significantly enriched motifs in relation to these infection-dependent splicing changes.

In any case, because so much of this paper, including the identification of lark as a regulator of interest, involves comparison of retained introns to this undefined set of introns, "spliced introns" absolutely must be defined clearly.

The authors emphasize a small but significant increase in average 5' UTR length but not more frequent and significant changes in 3' UTR length (which do not affect average length because they are about equally increasing and decreasing length).  A little more emphasis on the variability of 3' UTRs would be appropriate. Also, something might be said about the nature of the length changes in 3' UTRs. What fraction are due to changes in splicing? What fraction are due to changes in polyadenylation site use?

Specifics:
Fig. 5 - The greatest enrichment observed for SF1, HOW and CNOT4 and in "spliced introns." Depending on what "spliced introns" are, this could be of considerable interest.

Supplemental Fig. 2 (and elsewhere).  The analysis of transcript ratios is problematic because of the possibility of incomplete annotation.
Take the case of PSI. There are two local alternative splicing events. One is at the SNP itself, where a downstream splice site is used in transcripts 87072 and 87073 but not 87074. The other local AS event is an intron retention event well upstream. The problem is that there is no annotated transcript that retains the upstream intron AND uses the downstream choice at the SNP.  If the variant only affected splicing locally, changes in the estimation of the abundance of 7072 could result from changes in the abundance in the non-annotated transcript.  I'd like to see changes in the local splicing events separately reported; in any case, the authors should satisfy themselves that the reported changes are not an artifact of incomplete annotation.

I like the application of a diversity measure (Shannon entropy) to these results, and I accept that the effect is consistent and significant. However, the effect is also small; this should be made clear in the text.

Figs. 1b and 2b. Presentation of GO results is always problematic because of the need to depict three things: p values, enrichment and number of genes.  While I see the appeal of the semantic space representation, it fails to convey any of those precisely and does not even provide the actual GO categories. For example, there is one blue circle that is clearly the most significant of categories, and we aren't told what it is. Whether or not the authors revise this figure with a more informative summary, the gene ontology categories should be reported with p value, number of genes and enrichment in a table (which can be supplemental data).

Fig. 1c. These curves are clearly smoothed. Say so.

The plots in Fig. 2c and 2d communicate much less than they could, and the extreme smoothing of curves obscures the information being conveyed. The bars, which are not smoothed, presumably represent the distribution of sQTLs rather than the distribution of background variants; this should be stated. It would be much clearer if the background distribution were likewise presented without smoothing, perhaps as paired bars.  Also, Fig. 2d shows the position of sQTLs (and all variants) scaled to intron length. Because readers will be interested in the distribution of sQTLs relative to splicing signals (and the authors refer to this at ll. 204-205), it would be much more informative if the scale here were fixed relative to true length in nucleotides (which would necessitate breaking the plot into 5' and 3' splice sites).

**Authors Response**

**Point-by-point responses to the reviewers’ comments:**

**Reviewer #1:**

This manuscript looks at the changes to splicing that occur during the Drosophila gut immune response. This is to my knowledge entirely novel - there have been numerous studies on transcriptional responses to infection but these have tended to be restricted to transcript abundance. The results are striking, with extensive intron retention extending the length of 5'UTRs. The authors then convincingly link this to an RNA binding protein called Lark. Knocking-down or overexpressing this protein affects susceptibility to infection, suggesting this may be important in resisting infection. I found this to be an important advance in our understanding to the response to infection and splicing more generally.

We thank the reviewer for his/her appreciation of our work and are excited that he/she finds it relevant and interesting.

The manuscript leaves open the critical question of the function of intron retention in the 5' UTR. However, this is sensibly discussed and I am content this falls beyond the scope of this manuscript. I was also left wondering whether this was a general stress response or a more specific immune response, but again feel this can be left for the future. Infection in flies can cause quite dramatic changes to the gut tissues, so different infection regimes may alter the abundance of different cell types in the samples. It would be nice to have a few words describing how guts respond to this particular infection and the consequences of this for the results. In places the inclusion of natural genetic variation felt peripheral to the main narrative.

Indeed, general stress is a subset of the defense response to infection. We believe that many of the observed changes can be reproduced with other insults. One line of evidence is the similarity between *Ecc15* and *Pe* infection on the transcriptome. *Ecc15* is known to induce lower to no mortality in flies and thus less stress. Also to note, the transcriptome profiling is after 4 hours of infection, a time point where most of the major changes in the cell type abundance of gut tissues would not have occurred yet. Our conclusions from the presented analyses is that the the extent of splicing scales with the degree of infection, which implies degree of stress. Another reason to believe that this is a generalized stress response versus an immune response is that we do not observe enrichment of canonical immune genes among those affected by splicing at the transcript isoform level. We added one sentence in the discussion, which already discusses stress, to make this important point clearer (Line 428-430).

The concise description means it would be challenging to reproduce the analysis. Making scripts public can solve this.

We have now added the relevant scripts and data on Zenodo (doi: 10.5281/zenodo.3368857).

**Reviewer #2:**

In this paper, the authors have examined the effect of enteric infection on gene expression, including differential alternative splicing, in the Drosophila Genetic Reference Panel inbred lines. They find that while there are few differences in alternative splicing between resistant and susceptible lines, infection has dramatic effects on alternative splicing and reveals cryptic genetic variation in splicing (infection-dependent sQTLs). On average, post-infection transcripts tend to be longer, mainly due to 5' UTRs. These results are significant, and will interest anyone concerned about immunity or the regulation of splicing.

We thank the reviewer for his/her positive feedback and interest in our manuscript.

However, "extensive intron retention" (in the title) focuses excessively on one aspect of the observed phenomenon and is an overstatement.

We agree with the reviewer and have therefore removed the word “extensive” from the title, which, in our opinion, still adequately captures the most striking findings of our study.

There are many analyses here, and some are poorly described. The most important of these concerns the "spliced introns" comparison set. The identification of lark as a factor of interest is based on a comparison of retained and "spliced" introns (Fig. 5f).  I understand that "retained introns" is used here to refer to introns that are preferentially retained in response to infection. But what are the "spliced" introns? This set is used many places in the paper but never defined.  All introns are, by definition, spliced, so the term itself is not useful. The number of "spliced" introns and their properties also suggest that a specific definition is being used. Methods (ll. 599-600) states that introns that do not change significantly after infection were used as background in the RNA-binding motif analyses, so "spliced introns" must be a subset. One guess is that the "spliced introns" are those that do change significantly, but which are not retained intron events. These "spliced introns" would therefore be infection-dependent changes other than intron retention (i.e. alternative 5' splice site, cassette exons, etc.). Another possibility is that the "spliced introns" are those whose retention goes down following infection. This seems unlikely since this class would also be retained introns, and the same intron could be in different categories in different lines.  

We apologize if the definition of spliced versus retained was not clear in the original manuscript. It is true that all the introns are spliced to some degree, but our definition of retained and spliced introns has more to do with the infection-induced differences in intron retention (as estimated by delta PSI). In Fig. 4a, if delta PSI is less than 0, this means more splicing and vice versa. Now to define the subsets of introns that are spliced or retained, we used the following rule:

- For non-DGRP data: a retained intron is an intron that has a delta PSI > 0.2 and a Bayes factor >10. A spliced intron is an intron that has a delta PSI < -0.2 and a Bayes factor >10.
- For DGRP data: we do the same as above on a strain by strain basis. Then we defined retained introns as those that are observed in at least 5 strains. Similarly, spliced introns are those that have a significantly lower PSI in at least 5 strains. Since we show that intron retention is deterministic and reproducible between strains as well as infection types, if the same event is observed at least 5 times, then the expectation is that all other strains would behave in a similar way. For instance, among the 535 retained events that occur more than 4 times in the DGRPs, 510 are not spliced, 13 are spliced in one strain, 11 are spliced in two strains, and one is spliced four times. This rationalizes the implemented threshold of 5.

We made changes in the text to make this clearer (lines 267-275) and hope that the reviewer now finds this satisfactory.

Concerning the introns that do no change significantly, these are all introns that are not “retained” nor “spliced”, based on our definition. So the set of “spliced introns” does not constitute a subset. In other words, the total number of events = non-significantly changed + spliced + retained. The sequences of introns that did not significantly change after infection were used as background/control sequences when performing RNA-binding motif enrichment analyses. We adapted the Methods text accordingly (Lines 646-653)

Fig. 5f shows that the RNA-binding motif enrichment in set of "spliced introns" is, in general, much greater. We should be told more about this set of introns and the location of these more-significantly enriched motifs in relation to these infection-dependent splicing changes.

We have performed motif scanning using FIMO for all RNA binding motifs. As expected from their longer lengths and the motif enrichment analyses, there are many more motifs that map to these introns. When we explored the location of these predicted binding sites with respect to the introns’ respective 5’ splice sites, we found that motif occurrence is biased toward the 5’ splice site. We have added two panels to Supplementary figure 5 (d-e) to report these results (see also lines 329-336 in the Results Section) and added a brief description of our analysis to the Methods section (647-653).

Regarding the “introns themselves”, please see our response to the first point of “Specifics” below.

In any case, because so much of this paper, including the identification of lark as a regulator of interest, involves comparison of retained introns to this undefined set of introns, "spliced introns" absolutely must be defined clearly.

Please see our response to this issue above.

The authors emphasize a small but significant increase in average 5' UTR length but not more frequent and significant changes in 3' UTR length (which do not affect average length because they are about equally increasing and decreasing length).  A little more emphasis on the variability of 3' UTRs would be appropriate. Also, something might be said about the nature of the length changes in 3' UTRs. What fraction are due to changes in splicing? What fraction are due to changes in polyadenylation site use?

We thank the reviewer for bringing up this point since we have now realized that we did not properly discuss it in the original manuscript. First, we do see differences in effective lengths of 3’ UTRs, but they are actually lower than the permutation-based null distribution. Thus, it seems that 3’UTRs are actually more stable than expected, based on our strategy. To answer the second question about the proportional changes due to differential polyadenylation site use versus splicing, we devised a strategy to classify multi-isoform genes into these two groups. We used simple rules to decide whether annotated 3’ UTRs of that gene (1) can contribute to diversity in the first place, (2) have the same number of exons, and (3) share a common start position and different end position. Using this strategy, we obtained three groups of genes (out of a total of 3733 genes with more than one transcript):

- Splicing: Genes for which splicing can affect the 3’ UTR length (n = 387)
- Alternate3Poly: Genes for which an alternate choice of polyadenylation site can affect the length of the 3’UTR (n = 1138)
- No annotated diff / Ambiguous: Genes with either no difference in isoform length or ambiguous classification (n = 2208)

For example, if a gene has more than one isoform, we would first check if it has diversity in 3’UTR lengths of the different isoforms. Then we would look at those 3’UTRs and check if they are encoded by the same number of exons. If not, then effective length of these 3’UTRs would likely be affected by splicing. If all the transcripts’ 3’UTRs have the same number of exons, and these exons share the same start position but different end position, we put the gene in the “Alternate3Poly” category.

We then broke down the 3’UTR effective length changes as in Fig. 3b by UTR class. The results show that there are no differences between the groups that increase or decrease in length in terms of UTR class. In addition, we do not see any over-representation of one group over the other in terms of directionality of effective length change. Finally, the expected ratio of Splicing:Alternate3Poly (~1:3) is maintained in the genes that increase, decrease, and remain stable in effective 3’ UTR length. We added a panel depicting these changes (revised Supplementary Figure 3d) and added a short description of these results in the revised manuscript (lines 231-232, and 617-632).

**Figure Legend**: Breakdown of 3’ UTR effective length changes by 3’ UTR class. Each gene with more than one isoform was classified into 3 groups, depending on how its isoform lengths may be affected by 3’ UTR changes. Alternate3Poly is the group of genes whose 3’ UTRs vary in 3’ polyadenylation site usage. Splicing is the group that is affected by alternative splicing. The remaining genes are either complex or ambiguous, or do not have diverse 3’ UTRs. The percentage is the percentage of all genes that increases, decreases, or does not change in effective 3’ UTR length.

***Specifics:***
Fig. 5 - The greatest enrichment observed for SF1, HOW and CNOT4 and in "spliced introns." Depending on what "spliced introns" are, this could be of considerable interest.

Indeed, the spliced introns feature a stronger enrichment for RNA binding proteins than retained introns. In addition, we found that infection-driven spliced introns (based on our definition), behave like canonical introns in that (as shown in Fig. 5), compared to retained introns, they are longer, have stronger splicing signals and sequences, lower GC content and lower PolII occupancy. These thus represent introns that, in our opinion, undergo splicing efficiently even during infection or stress. We did not focus on these spliced introns because we were interested in the intron retention phenomenon, but they are certainly still interesting to explore further. We hope that the introduced definitions (lines 267-275) that address the reviewer’s earlier point about definitions of the different intron groups renders the message clearer now.

Supplemental Fig. 2 (and elsewhere).  The analysis of transcript ratios is problematic because of the possibility of incomplete annotation. Take the case of PSI. There are two local alternative splicing events. One is at the SNP itself, where a downstream splice site is used in transcripts 87072 and 87073 but not 87074. The other local AS event is an intron retention event well upstream. The problem is that there is no annotated transcript that retains the upstream intron AND uses the downstream choice at the SNP.  If the variant only affected splicing locally, changes in the estimation of the abundance of 7072 could result from changes in the abundance in the non-annotated transcript.  I'd like to see changes in the local splicing events separately reported; in any case, the authors should satisfy themselves that the reported changes are not an artifact of incomplete annotation.

We thank the reviewer for his/her keen insights and thoroughness. We agree that incomplete annotation may cause analytical problems, however, we believe that dealing with this issue would require intensive re-characterization of the *Drosophila* transcriptome in order to refine the available annotations. For this study, we relied on the excellent work performed by the Wittkopp and Graveley labs (McManus et al., 2014) that used female flies to characterize intron retention events, among other splicing events.

One dilemma we faced is that the splicing event annotations do not always map to introns in the transcript-level Ensembl annotations. Even if the annotations align well, mapping from individual events to transcript isoform abundances is non-trivial: the same event can be shared by multiple transcript isoforms and the integration of the effect of multiple events (intron retention or other) is not possible with short-read NGS data. We would have preferred to provide a better answer to the reviewer’s question, however, we believe that this is very challenging and out of the scope of this manuscript. That said, it is certainly an issue that can be addressed in the future, for example through integration of our and other labs’ data including those stemming from other sequencing technologies such as long-read sequencing.

The transcript ratios, sQTL, and other analyses based on isoforms are thus incomplete, given the limitations in annotation. However, we believe these analyses are still informative. Knowledge as to whether or not there is a possible fourth (or more) transcript of PSI, for instance, would certainly provide us with better precision, but would unlikely change the fact that there is a genetic variant affecting splicing for that specific gene. For this study, we were interested more in the latter. Please note that we have in fact performed sQTL analyses on individual intron retention events because it is technically feasible, but we do not believe that including these data would add value to the manuscript since it would complicate the story and dilute the core findings. Instead, we addressed this issue in the results (lines 254-261) and discussion (lines 408-414). Nevertheless, we are willing to include these analyses in the manuscript if the reviewer deems it necessary.

I like the application of a diversity measure (Shannon entropy) to these results, and I accept that the effect is consistent and significant. However, the effect is also small; this should be made clear in the text.

We changed the wording in the text: “general increase in diversity” became “small but consistent”.

Figs. 1b and 2b. Presentation of GO results is always problematic because of the need to depict three things: p values, enrichment and number of genes.  While I see the appeal of the semantic space representation, it fails to convey any of those precisely and does not even provide the actual GO categories. For example, there is one blue circle that is clearly the most significant of categories, and we aren't told what it is. Whether or not the authors revise this figure with a more informative summary, the gene ontology categories should be reported with p value, number of genes and enrichment in a table (which can be supplemental data).

We have included supplementary tables (Supplementary data 2) that include the GO analysis results to provide more information to the reader.

Fig. 1c. These curves are clearly smoothed. Say so.

These curves were generated with R’s base function density(). We added a sentence in the legend of Fig.1. “The densities were obtained using R’s base density function.” We have made the underlying analysis code also available.

The plots in Fig. 2c and 2d communicate much less than they could, and the extreme smoothing of curves obscures the information being conveyed. The bars, which are not smoothed, presumably represent the distribution of sQTLs rather than the distribution of background variants; this should be stated. It would be much clearer if the background distribution were likewise presented without smoothing, perhaps as paired bars.  Also, Fig. 2d shows the position of sQTLs (and all variants) scaled to intron length. Because readers will be interested in the distribution of sQTLs relative to splicing signals (and the authors refer to this at ll. 204-205), it would be much more informative if the scale here were fixed relative to true length in nucleotides (which would necessitate breaking the plot into 5' and 3' splice sites).

We have changed Fig. 2d and represented all data now as histograms. Also, we made a new figure (Supplementary Fig. 2e) which contains richer information. The metaplots in the new figure provide two insights: position relative to the two splicing signals of all sQTLs, as well as the positions of sQTLs that actually lie within an intron versus those that do not.

**Second round of review**

**Reviewer 2**

This revised manuscript is largely acceptable. However, there is one essential revision, a critical point of nomenclature.

Alternative splicing events are generally classified into types, including: cassette exons, mutually exclusive exons, alternative 5' splice sites, alternative 3' splice sites, and retained introns.  Any cases where there are two alternative that differ by an included intron are placed in the "retained intron" class, independently of the value of PSI or whether intron retention is increased or decreased by a particular treatment. Thus, the category "spliced introns," which plays a critical role in this paper is (now) defined as a class of retained intron.  Thus, the authors contrast two classes of retained intron, calling them "spliced introns" and "retained introns." This is extremely confusing and cannot be allowed.
The authors MUST find alternative terms for BOTH sets, define them clearly, and use those terms consistently.

**Authors Response**

**Point-by-point responses to the reviewers’ comments:**

We have addressed the outstanding issue that reviewer #2 had raised concerning the naming of intron retention events, which we agree could have led to confusion. Specifically, introns that were called “retained” in the previous version are now referred to as “introns with increased retention”. Similarly, the “spliced” introns of the previous version are now referred to as “introns with decreased retention”. We believe this addresses the semantic issue that was raised by reviewer #2.
